# ECM-derived biomaterials for regulating tissue multicellularity and maturation

**DOI:** 10.1016/j.isci.2024.109141

**Published:** 2024-02-06

**Authors:** Ali Smandri, Maimonah Eissa Al-Masawa, Ng Min Hwei, Mh Busra Fauzi

**Affiliations:** 1 Centre for Tissue Engineering and Regenerative Medicine, Faculty of Medicine, Universiti Kebangsaan Malaysia, Kuala Lumpur 56000, Malaysia

**Keywords:** Biological sciences, Tissue engineering

## Abstract

Recent breakthroughs in developing human-relevant organotypic models led to the building of highly resemblant tissue constructs that hold immense potential for transplantation, drug screening, and disease modeling. Despite the progress in fine-tuning stem cell multilineage differentiation in highly controlled spatiotemporal conditions and hosting microenvironments, 3D models still experience naive and incomplete morphogenesis. In particular, existing systems and induction protocols fail to maintain stem cell long-term potency, induce high tissue-level multicellularity, or drive the maturity of stem cell-derived 3D models to levels seen in their *in vivo* counterparts. In this review, we highlight the use of extracellular matrix (ECM)-derived biomaterials in providing stem cell niche-mimicking microenvironment capable of preserving stem cell long-term potency and inducing spatial and region-specific differentiation. We also examine the maturation of different 3D models, including organoids, encapsulated in ECM biomaterials and provide looking-forward perspectives on employing ECM biomaterials in building more innovative, transplantable, and functional organs.

## Graphical abstract

**Figure:**
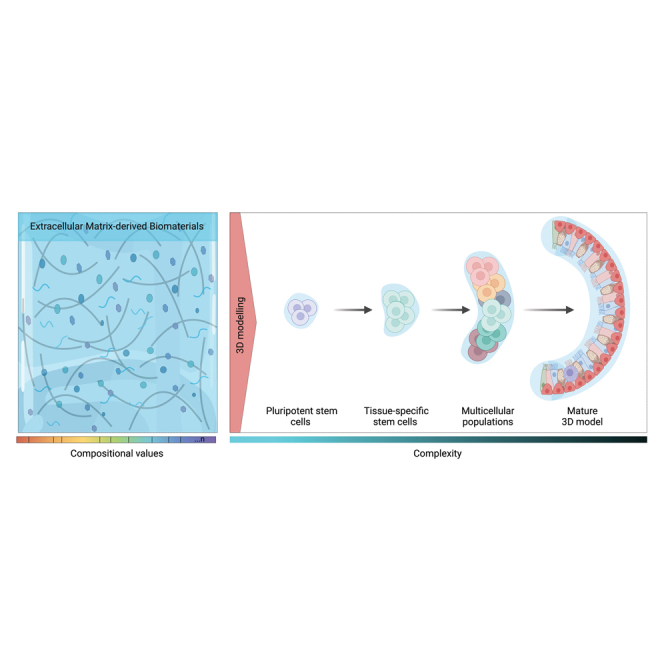

## Introduction

Current advancements in 3D modeling focus on replicating the early stages of human development by incubating potent stem cells in instructive microenvironments to facilitate fate specifications and dimensional expansions toward different developmental trajectories. Over time, the generated multilineage cells develop into distinct tissue-specific architectural configurations, forming high-fidelity human-like 3D models. These 3D models can be refined and steered to undertake self-guided or shape-guided morphogenesis to mimic the physiological complexity of human tissues.^1^^,^^2^^,^^3^^,^^4^^,^^5^ However, minimal success was achieved in bringing multicellularity and function to current stem cell-derived 3D models, mainly due to stem cell induction protocols incapable of stimulating organ-level multicellular populations and stem cell-derived cells lacking maturation.

Multicellularity and maturation become more of an issue with increasing system complexity. 3D models are the most challenging to mature as biomaterials get involved. Besides their limited compositional values and the lack of instructive signals necessary to initiate sequential developmental events, traditional biomaterials fail to guide or allow stem cells to undergo progressive differentiation into tissue-specific multicellular populations. Biomaterials with high density and fiber content can also undesirably affect cell morphology, interrupt collective cell migration, and disturb leader cell matrix infiltration.^6^^,^^7^^,^^8^^,^^9^^,^^10^ Such downsides obstruct the manifestation of comprehensive cell responses to instructive signals and reduce the chances of cell and tissue maturation. Specifically, biomaterials may introduce additional complications for cells to overcome, regardless of their assistive roles during morphogenesis. Consequently, it is still necessary to develop non-fibrous, multi-compositional biomaterials capable of preserving stem cells’ potency, encouraging *in situ* differentiation, and boosting cell expansion and maturation “in one go” for undisturbed *in vitro* morphogenesis.

In this review, we present extracellular matrix (ECM) biomaterials as a niche to generate different tissue-specific cell lineages of embryonic and adult tissues. We also focus on the role of region specificity in determining cell fate and phenotypic turnovers. Building on that, we roll into the concept of cell and tissue maturation and the recent applications of ECM biomaterials to enhance the maturation of multicellular systems and 3D models previously reported to lack maturation. The review also provides looking-forward perspectives on the utilization of ECM biomaterials, as superior to collagen-, laminin-, and Matrigel-based biomaterials, for the design of mature human-like 3D models.

## ECM biomaterials compositional values

**Figure 1:**
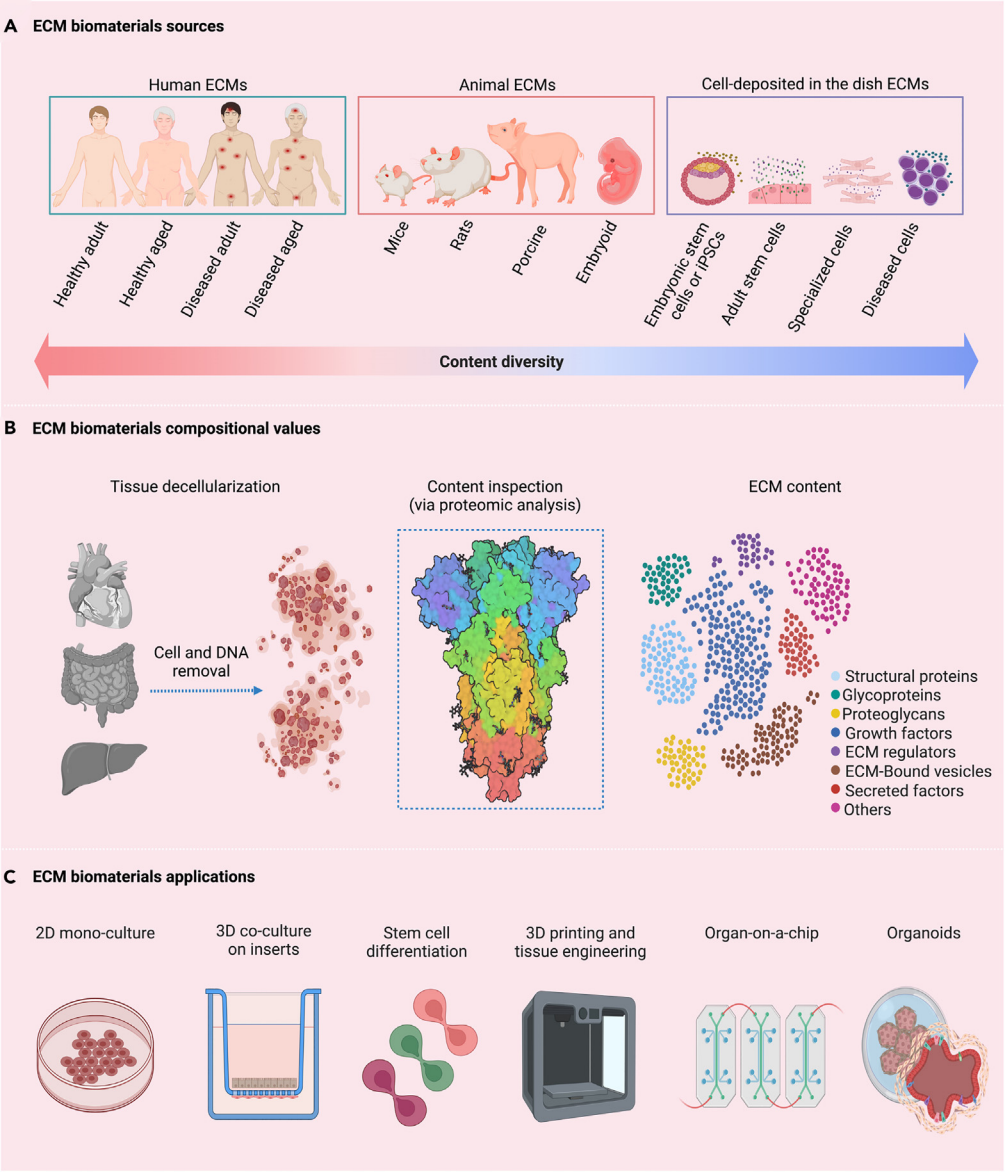
The extracellular matrix represents the space fabric of the biological life of cells, forming strong lifelong adhesive bonds, controlling cells’ behavior and function, and mediating cells’ communication with their surroundings (A) Tissues can be obtained from healthy and diseased tissue sources or collected from embryonic and adult cell cultures. (B) Collected tissues are decellularized using chemical, physical, enzymatic, and apoptosis-assisted approaches, causing prominent cell necrosis or stimulating programmed cell death while maintaining intact ECM structure and content (*decellularization protocols are not covered in this review*). (C) Decellularized ECMs can be materialized using different chemistries to form powders, hydrogels, or liquid supplements, standalone or combined with traditional biomaterials for various applications, including their leading roles in building current 3D models.

Living cells are programmed biological entities that respond to input signals and generate intracellular and extracellular outputs, including protein deposition and assembly into a unique tissue-specific ECM. The ECM plays significant roles as a hosting microenvironment for diverse cell lineages, such as preselecting development boundaries during embryogenesis, providing structural support to residing cells, and serving as a reservoir of thousands of proteins, growth factors, bound extracellular vesicles, and other cell-secreted factors, mediating cellular activities and ensuring tissue hemostasis.^11^^,^^12^^,^^13^^,^^14^^,^^15^^,^^16^ The ECM structure is cell controlled and maintained, varies from tissue to tissue, and undergoes dynamic changes with aging and disease.^17^^,^^18^^,^^19^ The ECM also serves as a stem cell niche, an exclusive microenvironment that guards and controls adult stem cell potency and fate specification.^20^^,^^21^ The development of ECM mimicries, which offers such a broad spectrum of values to cellular life (Figure 1), has captured significant interest in tissue engineering and regenerative medicine.

The primary structural components of the ECM are the fibrous proteins collagen, fibronectin, elastin, laminin, and others, and the glycosylated filler proteins proteoglycans and glycosaminoglycans (found in the ECM interstitial space in a hydrated gel-like form) give the ECM its unique 3D architecture and provide a framework for the localizing cells.^22^^,^^23^ Each tissue has definite regional ECM compositions and structures that serve its physiological needs and location.^24^ The growth of using ECM biomaterials led to considerable advances in decellularization strategies to free the ECM from all the cellular remnants and preserve the inherited compositional values. For example, over 1600 matrisomes (a broad array of “parts list” of all soluble ECM-related proteins^25^) were identified in porcine intestinal decellularized ECM, 130 of which were ECM matrisome proteins, and 619 were extracellular vesicles-related matrisomes bound to the ECM.^26^ Canine chorion placenta decellularized ECMs were enriched in 52 proteins, including structural proteins, growth factors, and remodeling enzymes, besides proteins related to skeleton structure, collagen catalyzing, and germ layer development.^27^ Although tremendous literature has emerged reporting the high count of unique compositional values in decellularized ECMs,^28^^,^^29^^,^^30^^,^^31^^,^^32^^,^^33^^,^^34^^,^^35^^,^^36^^,^^37^^,^^38^^,^^39^^,^^40^^,^^41^ understanding the precise mechanism of action of the content of ECM biomaterials has not been sufficiently explored.

Cells release a heterogeneous group of anucleated particles bound by a lipid bilayer called extracellular vesicles (EVs). EVs are broadly categorized into exosomes (formed through the endocytic pathway) and ectosomes (formed at the plasma membrane),^42^ ranging from 50 to 1000 nm. Once considered cellular waste, these vesicles and particles were found to transfer bioactive cargos such as RNA, proteins, enzymes, transcription factors, and lipids, partake in cell-cell communication, induce phenotypic changes in recipient cells, and mediate various physiological and pathological processes.^43^ While early studies focused on EVs released into biological fluids and culture media, ECM- or matrix-bound extracellular vesicles (MBVs) still need further exploration. MBVs are a subset of EVs deposited and stored in the ECM and may have a different cargo (and possible functions) than fluid-released EVs.^44^ MBVs are immobilized and tightly attached to the fibrous structures of the ECM and released upon its degradation.^45^ New evidence suggests that MBVs can traverse the ECM pores.^46^ MBVs are increasingly recognized as a ubiquitous and integral part of the ECM and a substantial contributor to its function.^47^ MBVs have been demonstrated to mimic the functional properties of the ECM from which they were extracted. For example, MBVs separated from decellularized ECMs (i.e., porcine urinary bladder and small intestinal submucosa ECMs) promoted the pro-inflammatory (M1) to the anti-inflammatory (M2) phenotype transition, resembling the parent ECM effect on macrophages.^48^ The abundance of miRNA 125b-5p, miRNA 143-3p, miRNA 145-5p,^48^ and regulatory protein IL-33^49^ in those MBVs might be critical to this phenotypic transition. Similarly, MBVs derived from aged bone ECM exacerbated the “calcification paradox” that characterizes osteoporosis, wherein bone marrow mesenchymal stem cell (BMMSC) adipogenesis and vascular smooth muscle cell mineralization are induced. Enriched miR-483-5p and miR-2861 in MBVs might be implicated in this effect.^50^

Since multiple reports revealed a substantial role of tissue- or cell-specific EVs in promoting stem cell lineage-specific differentiation, exploring such important roles of MBVs could be promising. For instance, β-cell-derived EVs could enhance induced pluripotent stem cell (iPSC) pancreatic endocrine differentiation, generating functional β-cells *in vitro* and *in vivo*.^51^ Likewise, sequential administration of EVs collected from embryonic stem cells differentiating into cardiomyocytes enabled efficient “direct lineage conversion”^52^ of fibroblasts into functional cardiomyocytes (efficiency >60%) without passing through the pluripotency stage.^53^ Interestingly, the difference in miRNA content between chondrocyte ECM-derived MBVs of the growth zone and their miRNA content of the resting zone in the culture plates could represent the difference in the differentiation and maturation stages of these development zones.^54^ Such evidence may allude to MBVs’ role in ECM-mediated differentiation and maturation, as discussed later in this review. MBVs contribute to ECM bioactivity and carry tissue-specific instructive factors and distinct functions that may differ from liquid-phase EVs. Discovering novel types of MBVs and understanding their content and roles in different tissues will be crucial for deciphering the mechanism of the ECM functionality in driving stem cell multilineage differentiation and maturation.

## ECM biomaterials for spatial stem cell differentiation

The early embryonic stage of gastrulation involves generating three germ layers: ectoderm, mesoderm, and endoderm, which create more specialized stem/progenitor cells that progressively differentiate and evolve into specialized organ-specific multicellular systems.^55^ Intrinsic and extrinsic signals mediate this downstream stem cell fate specification.^56^^,^^57^ The extrinsic signals regulate the activation of lineage-determining intrinsic signals (a set of transcription factors, TFs), where TFs initiate stem cell differentiation, select the lineage (the overexpressed TF specifies the lineage^57^), and form a committed cell, acting in a cascade-like manner.^58^^,^^59^ Cell commitment (identity) is irreversible when the signals are removed.^60^ The need to precisely understand fate-specification mechanisms led to more research investigating the mediators of receiving lineage-determining extrinsic signals. Later research, however, found that stem cells reside in a stem cell-specific tissue-localized microenvironment called the “niche.” The niche regulates stem cell interactions with the surrounding environment and provides maintenance.^21^^,^^61^ The niche also protects stem cell populations,^62^ controls their quiescence and activation,^63^ allows in stem cells, and sends out daughter progenitor cells born during asymmetric stem cell division.^64^ Current understanding also suggests that stem cells can “architect” their niche^65^ by localizing on basement membranes and harnessing niche-building cells, binding proteins, blood vessels, and sensing neurons.^66^

**Figure 2:**
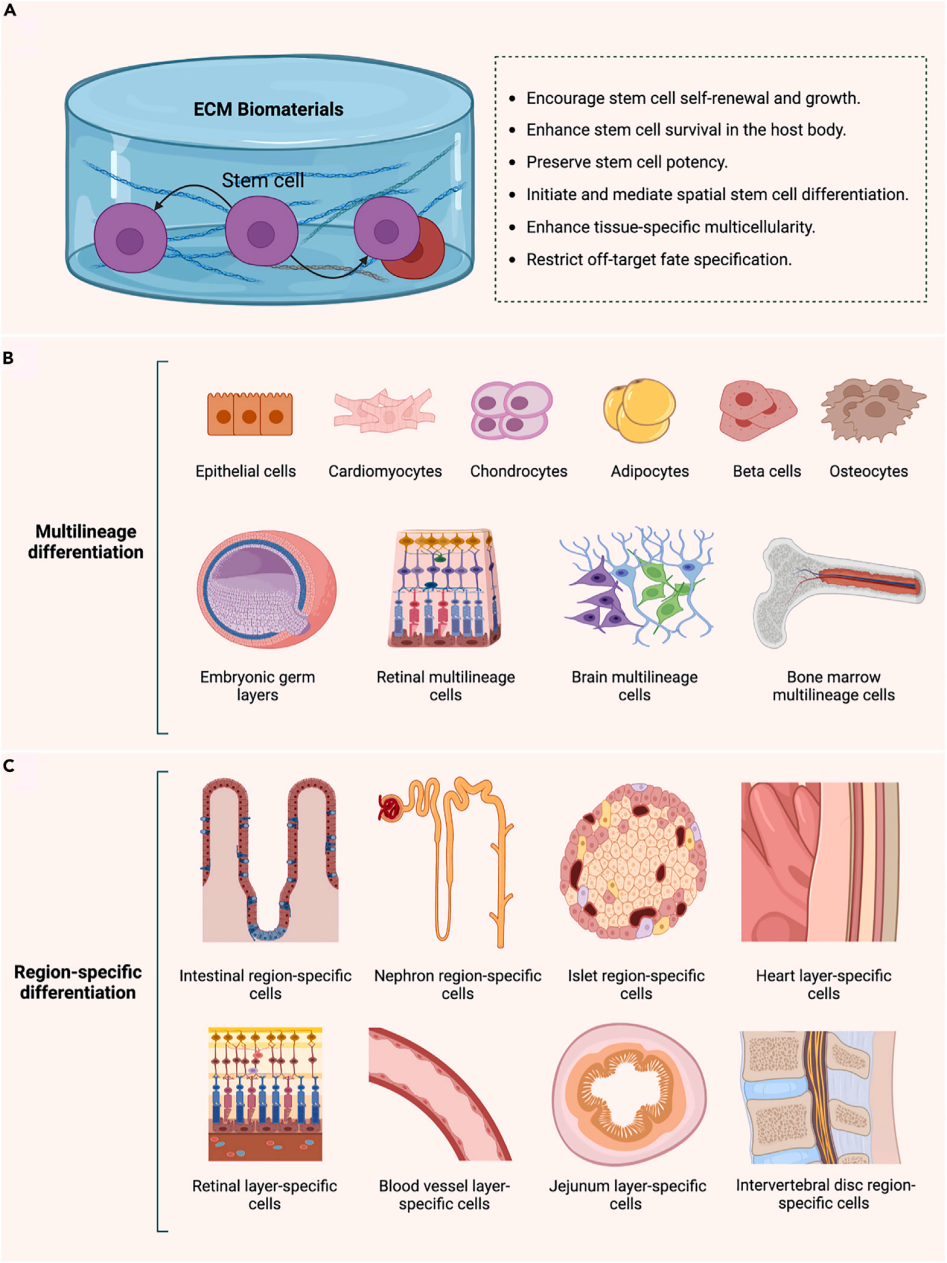
ECM-derived biomaterials as alternatives to stem cell niche (A) Maintaining stem cell potency is an essential role of stem cell niche, preserved in ECM biomaterials, and is needed in current 3D models. Such a role helps avoid the instant differentiation of a whole stem cell population, which can lead to incomplete morphogenesis and the absence of some cell lineages meant to occur after certain developmental events. ECM biomaterials were reported to maintain the potency of many stem cell types, including ESCs,^72^ iPSCs,^72^^,^^73^ HSCs,^74^ gastric stem cells, and intestinal stem cells.^75^ (B) ECM biomaterials used to encapsulate stem cells enable fate specification into many lineage-specific and multilineage cell types. (C) ECM biomaterials also contain region-specific niche signals suitable to induce stem cells to generate precise cell lineages with high region specificities, such as developing a diverse array of multiregional kidney nephron cells, including podocytes, glomerular capillaries, glomerular mesangial cells, proximal and distal renal tubules, the loop of Henle cells, renal collecting duct cells, and cuboidal cells.^76^^,^^77^^.^

Finding alternatives to the “niche” is one of the everlasting challenges impeding stem cell therapy and 3D modeling. This enduring challenge is underscored by a range of ongoing issues, including the vanishing of stem cell population following *in vivo* implantation,^67^^,^^68^ inadequacies in stem cell differentiation protocols,^69^^,^^70^ the undesired differentiation of a whole stem cell population into a lineage-specific population (the lack of fate regulators and stemness caretakers, resulting in a “loss of potency”),^71^ and the difficulty in stimulating “spatial fate specification” in both *in vitro* and *in vivo* microenvironments, indicating the lack of stem cell niche-mimicking microenvironments. Therefore, developing multifunctional biomaterials to facilitate stem cell survival, preserve stemness and self-renewal, provide essential tissue-specific differentiation enhancers or off-target silencers, and mediate safe and efficient fate choices into region-specific multicellular populations is necessary (Figure 2). The following two sections briefly discuss the use of ECM biomaterials to differentiate stem cells into tissue-specific cell lineages while serving as an engrafting niche-like microenvironment ready for transplantation or disease modeling.

### Lineage-specific and multilineage fate specifications

Biomaterials derived from cell-deposited and tissue-derived ECMs can spatially stimulate the differentiation of stem cells into lineage-specific populations, reported to surpass the capabilities of supplemented differentiation mediums and chemically induced differentiation protocols. Examples of this approach include developing osteoblasts, chondrocytes, adipocytes, and other cell lineages by culturing mesenchymal stem cells (MSCs) on cell-deposited ECMs.^78^^,^^79^^,^^80^^,^^81^^,^^82^^,^^83^^,^^84^ Similarly, pancreatic β-cells have been generated from human embryonic stem cells (hESCs) cultured on rat pancreatic β-cell-deposited ECMs.^85^ Beating cardiomyocytes were also derived from hESCs cultured on porcine heart ECM biomaterials.^86^ Nail epithelial cells were obtained from BMMSCs cultured on human digits-derived ECM scaffolds.^87^ Furthermore, corneal epithelial cells have been generated from hESCs cultured on human cornea-derived ECM biomaterials.^88^ These examples represent only a fraction of the extensive literature demonstrating the capacity of ECM biomaterials to induce stem cell lineage-specific differentiation *in situ* without differentiation inducers or supplementations. Developing biomaterials with a scent (small concentrations) of the ECM can enhance their functionality toward stem cells and generate functional tissues with representative cell types.

The use of pluripotent stem cells, such as ESCs and iPSCs, has been a driving force in the cutting-edge advances of regenerative medicine. iPSCs are widely adopted and can be derived by reprogramming patient somatic cells. They are then used to generate diverse organ-specific multilineage cells for studying patient-specific diseases and developing 3D models. However, the challenge lies in developing multicellular populations of cells from pluripotent stem cells in an event-driven manner in a 3D microenvironment. Thus, encapsulating pluripotent stem cells in ECM biomaterials is suggested to preserve their potency and ensure progressive fate choices for complete and well-driven morphogenesis.^73^ For example, the embryonic multilineages of the primitive streak and mesoderm, epiblast and mesoderm, visceral endoderm, and neuroectoderm were generated by culturing mouse ESCs on decellularized murine ESC-deposited embryoid bodies for 6 days.^70^ Similarly, the three germ layers were developed by culturing mouse ESCs or human iPSCs (hiPSCs) on decellularized ESC-derived ECM embryoid bodies. In the same study, mouse ESCs and hiPSCs cultured on decellularized neural progenitor cell-deposited aggregates underwent efficient fate changes into neuronal lineages while maintaining high potency.^72^

Adult stem cells, also known as organ-specific stem cells, are distributed throughout the body and play a vital role in lifelong tissue maintenance and repopulation, replacing problematic, aging, or diseased cells with new cells. The differentiation of adult stem cells is significantly influenced by the physical and biochemical cues within the niche. Complications in the niche or the surrounding tissue can disrupt adult stem cells’ function and lead to severe diseases. One such complication is the changes in niche stiffness associated with aging, which have been shown to decrease the differentiation capacity of adult stem cells.^89^^,^^90^ However, in the context of adult stem cell transplantation, it is crucial to use niche-mimicking biomaterials to guide these cells into the required multilineage cells of the target tissue. ECM biomaterials have an excellent potential for such applications. For example, hematopoietic stem cells (HSCs) cultured on decellularized umbilical cord Wharton jelly-derived ECM (WJ-ECM) biomaterials could generate all bone marrow primary multilineage cells of myeloid, megakaryocyte, erythroid, natural killer cells, T cells, and B cells within 7 days. It was also found that WJ-ECM-derived biomaterials retained HSCs stemness, promoted colonization, and enhanced HSC megakaryocytic differentiation affinity and transmigration capacity,^74^ closely mimicking the niche function and can be a future replacement to diseased bone marrow niches. Likewise, human dental pulp stem cells cultured on human dental pulp ECM hydrogels generated dental multilineage cells of odontoblast, neurons, and vascular cells, enabling dentinogenesis-like growth within 4 weeks.^91^

Notably, the age of the harvested tissues for decellularization plays a crucial role in determining their capacity to induce “efficient stem cell differentiation.” For instance, day-10.5 decellularized embryonic murine dorsal pancreatic bud could induce epithelial progenitor differentiation into all pancreatic exocrine and endocrine multilineage cells within 7 days.^92^ Thus, early-age tissue-specific ECMs, utilized as a source of ECM biomaterials, may exhibit greater plasticity than aged tissue ECMs, enabling efficient pluripotent stem cell fate specification into the downstream multilineage cells of the target tissue.

### Region-specific fate specifications

The region from which the induction signals originate determines cell responses and commitments.^93^ For example, the differentiation of neural stem cells into various multilineage neural cells is governed by neurogenic regions like the postnatal brain subventricular zone, which serves as the neuronal stem cell niche.^94^ Likewise, epidermal stem cells give rise to all epidermal lineages, operating from their niche at the basal layer of the skin epidermis.^95^ Interestingly, using ECM biomaterials highlights the significance of region specificity in determining cell fate choices. In the context of using ECM biomaterials, human BMMSCs cultured on intervertebral disc regions of nucleus pulposus or annulus fibrosus ECMs differentiated into different region-specific cells, mediated by the ECM niche signals of each region.^38^ Culturing renal stem cells on kidney ECM biomaterials resulted in the generation of diverse renal multilineage cells, including proximal tubular cells, distal tubular cells, epithelial cells, and endothelial cells.^96^ This suggests a direct stem cell interaction with the niche of multiple nephron regions. Interestingly, when neuronal stem cells (NSCs) were cultured on heart ECMs, they were subsidiarily found to have generated a small population of cardiomyocytes.^39^ This phenomenon may explain the reduction in the generation of off-target cells in 3D models made of region-specific ECM biomaterial.^76^

Whole-organ recellularization also represents an exciting avenue to better understand region-specific-mediated stem cell differentiation. Although stem cell delivery to the niche remains challenging,^97^ upon attaching to the ECM of the acellular organ (decellularized), the infiltrated stem cells instantaneously undergo fate specifications into region-specific multilineage cells. For instance, when ESC- and iPSC-derived cardiovascular progenitor cells were utilized to recellularize murine hearts, they localized to various niches, including the ventricular wall, vessel cavities, and endocardial surface. In response to the niche signals, they differentiated into cardiomyocytes, smooth muscle cells, and endothelial cells populating the ventricular heart wall, left and right ventricles, ventricle septum, and the apex. Interestingly, the generated multilineage cells contributed to the restoration of heart function, exhibiting spontaneous contractions at 40–50 beats/min within 20 days^98^ Similar literature on recellularizing rat kidneys,^99^ porcine retinas,^100^ and mouse pancreas^101^ reported the generation of multilineage cells from culturing stem cells on region-specific ECMs, suggesting the capacity of ECM biomaterials to spatially initiate comprehensive region-specific stem cell differentiation.

Allowing the host body to recellularize tissues or ECM biomaterial-based scaffolds is another exciting approach to prove this ECM differentiation-induction capacity. During this process, stem cells and progenies traveling through the bloodstream respond to the niche signals and repopulate the whole acellular organ with region-specific multilineages. For example, human smooth muscle cells were seeded into polyglycolic acid polymer scaffolds and allowed to deposit a new ECM into the scaffold over 8 weeks. Subsequently, the scaffold was decellularized to create bioengineered human acellular vessels (HAVs), prepared for a phase 2 clinical trial involving human single-arm implantation. The clinical trial spanned 200 weeks, with 60 patients enrolled. Interestingly, host cells progressively repopulated the implanted HAV with vessel multilineages such as smooth muscle cells, endothelial progenitors, neural progenitors, luminal cells, and MSCs, creating a mature multi-layer vessel that self-healed after cannulation injury, functioning like regular blood vessel of the host body.^102^^,^^103^ In a recent ground-breaking study, researchers linked a part of an acellular porcine kidney to a partial nephrectomy-unsealed porcine kidney, enabling recellularization and integration of the acellular part with the host kidney. Within 28 days, the acellular kidney part was seamlessly integrated with the host kidney, and it was repopulated with region-specific multilineage cells, including glomeruli (podocytes, glomerular capillaries, and glomerular mesangial cells), vessels (smooth muscle cells), and renal tubules (proximal and distal renal tubules, renal collecting ducts, renal progenitors, and cuboidal cells). The integrated kidney showed active blood flow and filtrate processing like a normal kidney.^77^ The available literature suggests the prominence of region-specific ECM biomaterials to generate multilineage cells and add more regional precision to current 3D models.

To efficiently mirror the function and structure of human tissues, engineered tissues or transplants must exhibit a considerable representation of cell types of the target multicellular tissue. Thus, the generation of high tissue-specific multicellularity represents the initial step in building functional tissues for regenerative medicine applications. Next, derived cells must show a high capacity to undergo systematic changes and adaptations (guided by essential instructive signals and conditions), reach a high level of responsiveness and readiness, and demonstrate convincing tissue-level maturation prior to their use for disease modeling or transplantation.

## ECM biomaterials for tissue maturation

**Figure 3:**
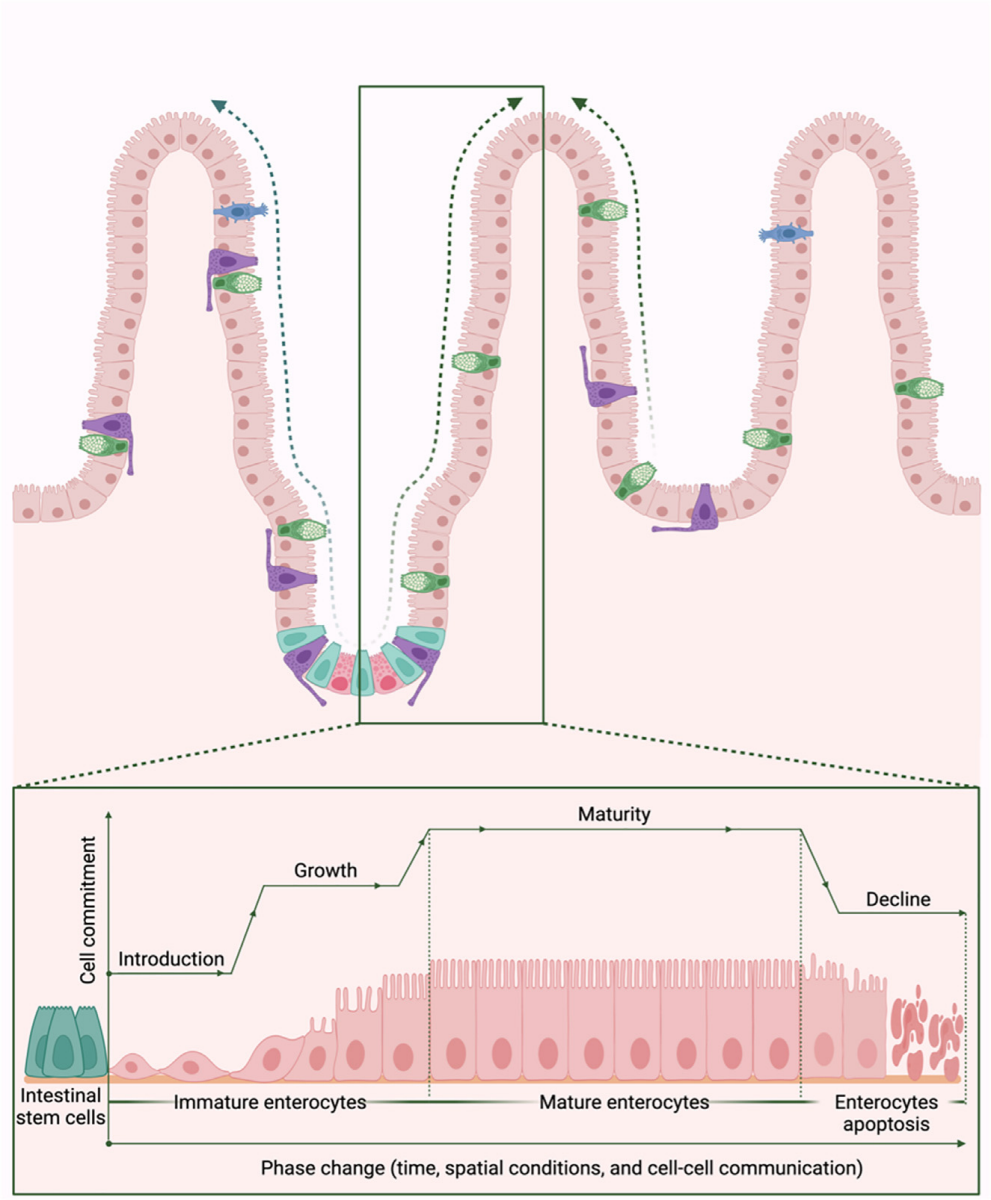
Following symmetric or asymmetric stem cell division, newly born naive cells endure gradual morphological and functional changes and embrace more tissue-specific commitments In this example, intestinal enterocytes generated at the intestinal crypts (intestinal stem cell niche) are pushed up to the top of the villus. Hereafter, enterocytes adapt unique apical-basal polarity (forming an apical membrane with tens of tiny villus to increase their absorption surface area) and start functioning on food digestion and absorption before they are discontinued at the villus top. However, the concept of phase changes and maturity is not exclusive to intestinal enterocytes but rather a general concept applicable to all cell lineages.

The emergent interdependence between cells and the ECM, acting as a regulator of receiving and forwarding instructive signals to all cell populations during cells’ life cycle, significantly contributes to tissue development and maturation. As a concept, a cell’s life cycle toward maturation includes the phases of introduction, growth, maturity, and decline (Figure 3). During the first two phases, cells appear irregularly shaped. Over time, an adaptive change in shape and size occurs, progressively allowing cells to fit into their large community like puzzle pieces. At this point, cells adopt specific alignments and polarities and undergo intracellular tissue-specific changes and adaptations that prepare them for high performance and to handle diverse challenges with full competence in the maturity phase. The maturity phase is the longest in the cell life cycle and can last for days (epithelial cells), weeks (skin epidermal cells), months (red blood cells), and years (neurons and heart cardiomyocytes) if the cell is competent enough to stay in the system. During this phase, mature cells exhibit superior and balanced metabolic activity, large-scale expression of mature genes and markers, higher bioenergetics and mitochondria amplification, improved ionic influx/efflux handling, and efficient ECM modeling and remodeling while performing routine tissue-specific functions. The maturity phase represents the threshold in the cell life cycle before their systemic withdrawal and replacement in the decline phase.

A mature enough cell or multicellular tissue model should exhibit the structural and functional characteristics of an adult human cell or tissue and respond to challenges (signals) in a manner similar to their human adult counterparts. For instance, when *in-vitro*-cultured pancreatic β-cells are challenged with low and high glucose levels for extended periods, they should display a mature glucose-responsive insulin-producing function, reflecting β-cell maturity.^104^ While understanding the time required for cells to mature is crucial, it is important to note that time alone does not guarantee cell/tissue maturation, and prolonged cultures may not always significantly influence maturation.^105^ What is exciting in the current advances is that researchers make the cells run a marathon to ensure their structural and functional maturity, particularly iPSC-derived cells.

As biomedical engineering is expediating the spin to developing tissue mimicry models, understanding cell maturation and identifying knowledge gaps are necessary. Cells grown in culture dishes may lose their maturity with passaging, and freshly derived stem cells (such as ESC- and iPSC-derived progenies and lineage-specific cells) may not be fully mature or “mature enough” to efficiently participate in 3D modeling campaigns.^106^^,^^107^^,^^108^ As a result, it becomes necessary to drive immature cells toward maturity using physical and biochemical approaches before employing them in tissue modeling or any other application (reviewed wonderfully by Alvarez-Dominguez and Melton^109^).

From the physics perspective of maturation, creating a mature enough organotypic model will require mimicking the *in vivo* system entropy level (the causatives of the momentarily deterministic and the future probabilistic behavior of a system). Looking at this from the point that maturity is a predictor of the cell's capacity to adapt to system entropy and contribute to maintaining the momentum of the progressively increasing system entropy toward the awaiting probabilities (thresholds that will take place to trigger the multicellular system to perform mature functions). This understanding may convey that without putting together mature cell populations, compositional values, and influential spatial factors, the generation of the same level of entropy in any tissue-mimicking 3D model is unachievable. Instead, a new domain of probabilities (narrower or wider) will take place, bringing into existence a thoroughly different tissue with an incomparable entropy level to the entropy level of known tissues. *In vivo* implanting such tissue will result in new probabilities (perhaps more unknowns) unless the *in vivo* system domesticates the implanted tissue to a close enough entropy level by remodeling the matrix and guiding infiltrative stem cells to differentiate into the missing cell lineages, which is highly unlikely to happen, due to the high entropy level of *in vivo* systems and the specificity limitation of currently used biomaterials.

Interestingly, recently modeled systems have come close to fully recapitulating adult human tissues despite the looked-for progress in biomaterial selection and design. For example, under induced differentiation conditions, 3D aggregated stem cells grow into multilineage cells of spherical or irregularly shaped structures known as organoids. In due course, organoids undergo morphogenesis to look and function like adult tissues.^110^^,^^111^ Organoids are primarily derived from iPSCs and are mainly used in developmental biology, drug screening, and studying patient-specific diseases for regenerative and personalized medicine. Tremendous efforts are ongoing to bring organoids and their development processes to similar development processes of their *in vivo* counterparts. Despite introducing different maturation factors, such as proteins, chemicals, circulating hormones, and electrical signals at different stages of organoid development to transform fetal-like organoids into mature adult-like systems,^111^ fully maturing organoids *in vitro* remains challenging.^3^^,^^112^^,^^113^

Encapsulating aggregated cells in a 3D physical space with a soft microenvironment (preferably nonfibrous) supplemented with instructive signals is crucial for their differential development into organoids. Many polymer hydrogels, primarily Matrigel derived from decellularized ECM secreted by Engelbreth–Holm–Swarm mouse sarcoma cells, have been used for this purpose. Although Matrigel has contributed to numerous successful organoid models by providing major ECM proteins and signals,^114^ it also comes with certain drawbacks, including its uncertain mechanism of action, batch-to-batch variation, xenogeneic contaminants, and the lack of tissue-specific biochemical signals required for stem cell multilineage differentiation.^114^^,^^115^ ECM biomaterials, while sharing some of the drawbacks of Matrigel, present a more inclusive and diverse alternative.^75^^,^^76^^,^^92^^,^^116^^,^^117^ The following sections represent the limited literature on using ECM biomaterials to mature organ-specific models and organoids.

### Nervous system multilineage maturation

hiPSC-derived neurons exhibit slower maturation than neurons of other species.^118^ Mature neurons develop structurally complex circuitry systems characterized by long axonal and dendritic branching trees capable of synaptic transmission and electrophysiological functions. The functional maturation of neurons is indicated by their ability to fire a series of action potentials in response to excitatory input signals, which requires efficient management of ionic influx/efflux and a conducive microenvironment. Additionally, neurons mature through interactions with other mature neuronal cells, including astrocytes, oligodendrocytes, and Schwann cells. Various biomaterials with different topologies and biochemical cues have been utilized to model neuronal circuit systems. Among these, ECM biomaterials have shown promise in promoting greater synaptic and postsynaptic functional maturation (reviewed intensively in Roth et al.^119^). For example, using fetal and adult porcine brain ECMs and 3D silk-collagen scaffolds, researchers improved the differentiation of hiPSC-derived NSCs and the maturation of NSC-derived cell lineages over six months. Fetal brain ECM specifically accelerated NSCs differentiation and resulted in the formation of a high-density axonal network of mature neurons and the maturation of star-shaped astrocytes. These cells also showed high structural integrity and mature secretion of chondroitin sulfate proteoglycans (CSPGs, an indicator of the maturity of neurons and astrocytes and critical factor in astrogliosis) compared to cells on silk-collagen scaffolds, which had unhealthy and clumped neuronal morphologies and overregulated CSPGs. In addition, the developing neurons on both fetal and adult brain ECM scaffolds had high levels of calcium activity (cell spikes and network bursts) and spontaneous burst activity.^120^ These findings underscore the potential of ECM biomaterials in promoting the functional maturation of iPSC-derived neurons and the formation of intricate neural circuitry.

Spinal cord ECM biomaterials were also used to build peripheral nervous system-resembling models. In a recent study, porcine spinal cord and peripheral nerve ECM hydrogels were used to induce NSC differentiation and guide the maturation of NSC-derived cell lineage. NSCs in spinal cord ECM hydrogels showed a 4-fold increase in differentiation into neurons compared to those in peripheral nerve ECM and collagen hydrogels. Spinal cord ECM hydrogels also appeared to improve the development and maturation of NSC-derived neurons’ synapses, as indicated by the upregulation of synapse organization-related genes and the activation of synapse formation-related Kyoto Encyclopedia of Genes and Genomes pathways. Excitingly, in an animal model of fully transected spinal cord injury, the injection of cell-free spinal cord ECM hydrogels into the gap allowed the host body to repopulate with NSCs. Penetrated NSCs showed signs of differentiation and contributed to some level of locomotor functional recovery within 8 weeks.^121^ Nevertheless, it is worth noting that even better outcomes might have been achieved if the transplanted ECM biomaterials were pre-populated with NSCs and allowed to mature *in vitro* before *in vivo* implantation.

Myelinating cells, such as oligodendrocytes and Schwann cells, play vital roles in neuron functional maturation by forming myelin sheaths around axons to propagate and accelerate action potential conduction. In the CNS, a single oligodendrocyte myelinates multiple axons of multiple neurons and has been found to improve neurons’ metabolic activity.^122^ Conversely, in the peripheral nervous system (PNS), a single Schwann cell myelinates a single axon of a multi-axon peripheral neuron. Impaired maturation (dedifferentiation) of myelinating cells can lead to many diseases, including common brain and spinal cord white matter disease.^123^ Finding methods to restore myelinating cells maturation *in situ* is still a medical hurdle. In a recent study, oligodendrocyte progenitor cells (OPCs) cultured on porcine optic nerve ECMs differentiated into 85% oligodendrocytes eventually developed into mature myelin-forming oligodendrocytes *in vitro*. Optic nerve ECM-loaded OPCs were developed into transplants to treat white matter defects in rat spinal cords *in vivo*. One month after implantation, the diseased rat models showed improved body weight support, a higher number of hind footprints (horizontal ladder-walking test), and reduced hindlimb errors compared to other treatment groups. The transplants also showed excellent integration with the host spinal cord, improving nerve regeneration and axonal remyelination and reducing inflammation and scar formation.^124^ Similarly, Schwann cells exhibited improved maturation and remyelination capacity when encapsulated in or cultured on ECM biomaterials.^125^^,^^126^

Less invasive approaches to treating neuronal cell diseases are preferable and can originate from ECM derivatives. For example, MBVs were isolated from decellularized ECMs and used as a neuroprotective and immunomodulatory factor to treat rat-induced retinal ischemia. Interestingly, ECM-derived MBVs prevented the axon degeneration of retinal ganglion cells, inhibited pro-inflammatory signaling by activating microglia and astrocytes, improved neuritogenesis, and preserved retinal function.^127^ ECM biomaterials derived from the CNS and PNS have shown great potential in the maturation of neurons, astrocytes, oligodendrocytes, Schwann cells, and microglia, facilitating the development of CNS- and PNS-mimicking models. In future research, it is essential to consider region specificity, mainly when modeling multiregional brain and spinal cord systems.

### Brain organoids maturation

**Figure 4:**
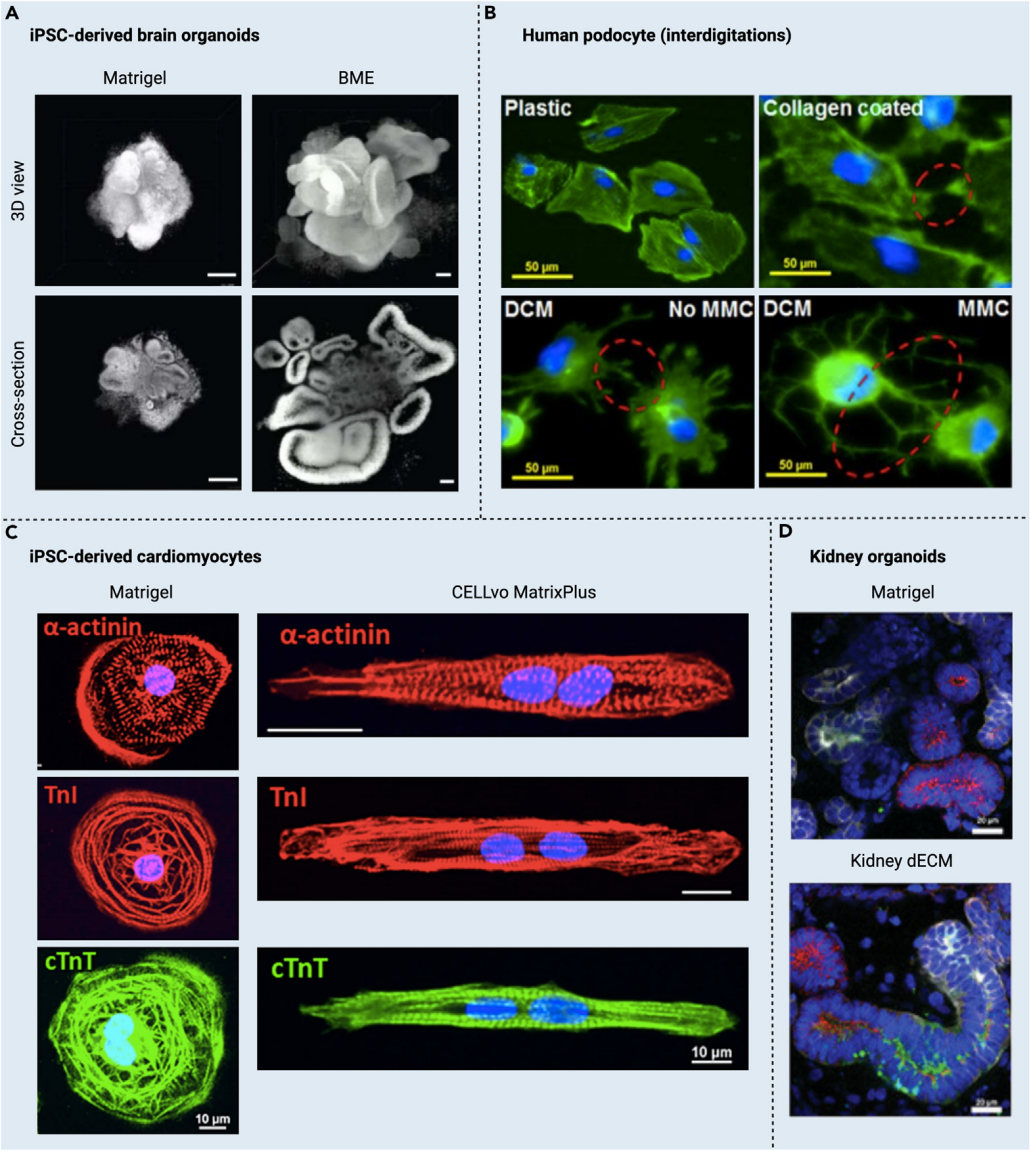
ECM biomaterials encourage the maturation of cells and organoids (A) BEM improves neurogenesis and organization of cortical layers in cerebral organoids encapsulated by Matrigel and brain ECM at day 75 (scale bars, 500 μm). Adopted from Cho et al.^131^ without changes. (B) Podocytes cultured (21 days) in DCM-coated plates developed interdigitating foot processes among neighboring cells, and the number of interdigits between podocytes was significantly higher (∗p < 0.0001, n = 15) in DCM-coated plates with macromolecular crowding (MMC) (scale bars, 50 μm). Adopted from Satyam et al.^133^ without changes. (C) Human ECM biomaterials (Matrix Plus) induce morphology changes compatible with adult-like phenotype. TnI, cTnT, and α-actinin immunostaining collectively indicate Matrix Plus induces rod-shaped morphology with the organization of sarcomeres parallel to the long axis of the cardiomyocyte (scale bars, 10μm). Adopted from Black et al.^116^ without changes. (D) Enhanced maturation of tubular structure of kidney organoids cultured on kidney dECM, represented by the efficient processability of drugs (dextran, green) along the distal tubule (E-cadherin, red) and proximal tubule (LTL, white) (scale bars, 20 μm). Adopted from Kim et al.^76^ without changes.

iPSC-derived brain organoids are multicellular brain clusters modeled to resemble human brain neural architectures and mimic their synaptic function and circuitry complexity. These models aim to study regional brain development and explore diverse genetic and medicinal therapeutics for brain diseases and neurodegenerative disorders. Indubitably, growing brain organoids is challenging and faces many hurdles, including their slow maturation (3–6 months), before they are considered reliable and brain-resembling.^128^^,^^129^^,^^130^ Evidence from the literature showed that providing brain organoids with necessary instructive signals and ensuring a suitable exchange of nutrients and oxygen using microfluidic systems can enhance the multilineage development of brain organoids and boost their structural and functional maturation. For example, in a mixture of hydrogels (brain ECM and Matrigel) and a dynamic microfluidic system, hiPSCs-derived cerebral organoids had better dimensionality, bigger diameters, and thicker laminin-rich basement membranes, and more volume and ventricle-like brain features (Figure 4). Many brain regions evolved within 30 days, resembling distinctive development stages with extensive neural networks and mature synaptogenesis. Various cell populations were present in these cerebral organoids, including neurons, astrocytes, radial glia, microglia, and Cajal-Retzius cells, with excellent electrophysiological functions such as sodium’s influx/efflux, postsynaptic currents, and multi-peak action potentials.^131^ The current literature suggests that brain ECM hydrogels offer unique region specificity, enabling the modeling of different brain regions such as the dorsal and ventral forebrain, hippocampus, hypothalamus, thalamus, cerebellum, midbrain, spinal cord, and others. These multiregional brain organoids can be further developed into multicellular brain assembloids or multi-regions-on-a-chip models^132^ to understand cross-regional brain development and model brain diseases with mature multilineage cells facilitated by region-specific brain ECM biomaterials.

### Cardiac multilineage maturation

Embryonic and fetal heart cardiomyocytes (CMs) endure massive changes in morphology, proliferation, gene expression, metabolism activity, bioenergetics, sensitivity to damage and apoptosis, and beating rate responsiveness while progressively growing into mature adult CMs.^134^ Adult CMs’ limited division capacity restrains their expansion in culture dishes. However, iPSCs induction to CMs and recent advances in “direct reprogramming” somatic cells (i.e., fibroblasts) to CMs increase their availability for cardiac tissue engineering. However, iPSC-derived CMs suffer the impediment of immaturity, rendering them ineffective for myocardial repair or disease modeling.^135^ The immaturity of CMs could result from the lack of synchronized physical and chemical cardiac mimicry microenvironment. Interestingly, early-stage iPSC-CMs (1-week post-differentiation) showed better responses to physical stimuli, as they have better plasticity over the adult- or late-stage iPSC-CMs, especially when introduced to spontaneous contractions^136^ or cyclic pulsatile medium flow (fluidic vertical pressure).^137^ Thus, maturing CMs has become more achievable recently, especially when using supporting cells (i.e., fibroblasts, MSCs), suitable encapsulation biomaterials,^138^ and proper physical stimulation systems.^139^^,^^140^ For example, under stretch and intensity training (electrical stimulation; 2 Hz on day 7 to 6 Hz on day 21, increased by 0.33 Hz per day), early-stage iPSC-CMs co-cultured with fibroblast in fibrin hydrogels exhibited remarkable maturity, similar to the maturity level of human adult CMs.^136^

Although ECM biomaterials were reported not to support CMs maturation, as CMs remained in the fetal developmental stages (reviewed by Scuderi and Butcher^141^), some recent work reported promising findings. Interestingly, hiPSC-CMs cultured on plates coated with decellularized perinatal stem cell-deposited ECM rapidly matured within 7 days, compared to hiPSCs-CMs cultured on Matrigel, as a booster of cell maturation (Figure 4).^116^ In another study, iPSC-CMs cultured in microtissues, supplemented with human heart ECM microparticles, experienced adequate maturation despite the short culture period, not co-culturing with fibroblasts, and not using a proper exercising system (used in earlier CM maturation examples). Many maturation signs were reported, including high collagen fibrils organization, improved beating rate (from ∼31.33 beats/min on day 11 to ∼49.0 beats/min on day 28), improved ultrastructure (sarcomeres length: 1.8 μm, organized Z-lines, A-bands, and I-bands, intercalated discs and gap junctions), high mitochondria density, and good calcium-handling activity.^142^ Thus, the new literature suggests heart ECM biomaterials as efficient biomaterials for designing heart-resembling models with mature CMs.

Most heart diseases take place due to aging. In one exciting example to evaluate ECM age reflexes on cell behavior and long-term maturation, mice heart ECMs at three age groups (young, adult, and aged) were used to coat glass surfaces prepared to grow young and aged iPSC-CMs. Interchangeably, young heart ECMs could promote the proliferation and division capacity of aged iPSC-CMs, while aged heart ECMs could not. Interestingly, aged heart ECMs were better at elongating young iPSC-CMs’ sarcomere length, a key player in contraction. Young and adult heart ECMs improved young iPSC-CMs’ cardiac function by increasing their beating frequency and velocity, maximum displacement, and calcium handling, showing great potential to mature young iPSC-CMs. Interestingly, under myocardial infarction-mimicking stress conditions, young iPSC-CMs cultured on young and adult heart ECMs showed better survival rates and less apoptosis and reactive oxygen species generation than those cultured on aged heart ECMs.^143^

Further research is needed to explore the use of heart ECM biomaterials to promote the maturation of CMs and construct mature heart models with representative multilineage cells. Leveraging heart ECM biomaterials in combination with iPSC-derived cardiac progenitor cells and iPSC-CMs to assemble a comprehensive range of heart multilineage cells and advance their differentiation and maturation through appropriate training systems is promising. To our knowledge, no study has reported the employment of heart ECM biomaterials to grow or mature heart tissues or cardiac organoids (a.k.a. cardioids), which can be a great area to explore.

### Pancreas multilineage maturation

The mature phenotypic interplay between α-cells, β-cells, δ-cells, and polypeptide cells in adult pancreatic islets allows them to respond effectively and rapidly to variant glucose levels in the blood. Irregularities in islet function lead to life-threatening complications, such as diabetes, which is the loss and dysfunction of insulin-producing β-cells. Current research enfolds around finding new methods and mimicry biomaterials to overcome iPSC-derived β-cells’ immaturity.^144^^,^^145^^,^^146^ Using pancreas ECM biomaterials was reported to promote insulin-producing cells (IPCs) and islet aggregates’ (spheroids/organoids) long-term survival and functional maturation *in vitro* and *in vivo*.^147^^,^^148^^,^^149^ For example, iPSC-derived islet aggregates (100–200 μm) encapsulated in porcine pancreas ECM hydrogels could mature within 7 days, evidenced by the expression of maturation markers (upregulation of CX36, GLUT1, PDX1, NKX6.1, and MAFA) and the efficient tolerance to low and high glucose levels (2.8 and 28 mM),^150^ suggesting the critical impact of native pancreatic niche signals on islet maturation. Wiring islets through a vascularization system can help improve cell-cell interaction, maximize cell diffusion and viability at the central points, and promote islets’ self-organization and functional maturation (reviewed intensively by Banerjee^151^). For example, co-culturing hiPSC-derived islet spheroids with human umbilical vein endothelial cell (HUVEC) in porcine pancreas ECM bioprinted scaffolds promoted their maturity and long-term responsiveness to glucose levels (day 4, day 6, day 8, and day 10), compared to their low responsiveness in collagen bioprinted scaffolds and 2D cultures,^152^ suggesting that vascularizing islet models encapsulated in pancreas ECM biomaterials is another key to ensuring their maturation and appropriateness for drug screening and disease modeling.

Interestingly, treating diabetic mice models with iPSC-derived IPCs-encapsulated porcine pancreas ECM-alginate hydrogels restored the mice’s normoglycemic conditions within 3 days. Herein, challenged mice models also showed mature and rapid responses to blood glucose (within 60 min), similar to non-diabetic models’ responses to high glucose levels. Moreover, pancreas ECM-alginate hydrogels promoted allogeneic IPCs’ long-term survival by suppressing the host immune system, indicated by the low count of neutrophils, lymphocytes, and M1 macrophages and the low M1/M2 ratio at the implantation site (week 4). Meanwhile, diabetic mice models treated with alginate hydrogel-encapsulated iPSC-derived IPCs or iPSC-derived IPCs only remained hyperglycemic. Noteworthy, non-encapsulated allogeneic iPSC-derived IPCs did not survive *in vivo*, where they could be screened out by the host immune system.^68^ Looking forward, using pancreas ECM biomaterials as hydrogels for islet encapsulation and maturation can bring the future of islet transplantation therapies handy. We hope to see more research questioning or examining the functional maturity of other multilineage cells within islets or islet aggregates (α cells, δ cells, and PP cells) to improve β-cells’ homeostasis and islet aggregates’ readiness for transplantation at large to push this promising therapy to the clinic.

### Pancreas organoids maturation

In the search for models that recapitulate the human pancreas, pancreatic organoids offer a great chance to study pathological conditions associated with diabetes and pancreatic adenocarcinoma. Recently developed islet organoids greatly mirror islet multilineage cells’ architecture and interrelated functions and hold great potential for disease modeling and transplantation. However, islet organoids lack functional maturity *in vitro*.^153^ As proof of the importance of native tissues to instruct multilineage differentiation in pancreas morphogenesis and maturation, embryonic murine dorsal pancreatic bud was harvested at E10.5 (embryonic day 10.5), wherein epithelial progenitor cells were isolated, and the remaining mesenchyme tissue was decellularized and used as ECM biomaterials to grow mature pancreatoids. All multilineage cells of the endocrine and exocrine pancreas were observed in pancreatoids on day 7, with a 22.42% population of insulin-positive β-cells and a 13.4% population of amylase-positive acinar cells. Pancreatoids had a 4.4 ± 1.6-fold larger size than Matrigel-embedded pancreas organoids and showed better self-organization, developing into two compartments for both acinar cells and β-cells. Although insulin-positive β-cells expressed many maturation factors and other endocrine markers at day 7, they could not develop mature glucose responses.^92^ However, it is too early to expect significant maturation within 7 days of organoid culture.

Pancreas ECM biomaterials can also be used to mature islet organoids. For example, in extensive proteomic analysis, rat pancreas ECM hydrogel was compared to Matrigel to detail the compositional differences. Pancreas ECM contained 42.3% extracellular proteins compared to 25.4% Matrigel extracellular proteins. iPSC-derived islet organoids cultured on a mixture of pancreas ECM and Matrigel showed a high expression of islet β-cell maturation markers (upregulation of PDX1, NKX6.1, MAFA, MAFB, and ARX) compared to islet organoids cultured on Matrigel only.^28^ A following-up study by the same group found that hiPSCs-derived islet organoids cultured on a mixture of pancreas ECM hydrogel and Matrigel contained all islet cell populations (α-cells, β-cells, δ-cells, and PP cells), which developed mature glucagon and insulin responses to low and high glucose levels (2 and 20 mM) similar to glucose-responsiveness of human pancreatic islets by comparison.^154^ Taken together, using pancreas ECM biomaterials in hydrogel form or hydrogel microparticles form^155^ side by side with other maturation methods like vascularization^156^ or microfluidic systems^157^ and training to tolerate glucose levels gradually can result in more mature islet organoids for transplantation. Furthermore, developing compartmentalized whole pancreas organoids or fusing exocrine and endocrine pancreas organoids into assembloids using region-specific pancreas ECM hydrogels to venture into understanding pancreas compartments and their interdependency^158^ is worth exploring.

### Liver multilineage maturation

Along with other multilineage of Kupffer cells (stellate macrophages), hepatic stellate cells, sinusoidal endothelial cells, hepatocytes, and cholangiocytes contribute to the main heavy-duty functions accomplished in the liver. Adult hepatocytes’ unique interlineage communication enables their competent albumin and bile production, lipid and drug metabolism, fatty acids uptake and processing, gluconeogenesis, and blood detoxification. Evidencing hepatocytes’ maturity *in vitro* is difficult, given the complexity and versatility of hepatocytes’ function. Despite the high qualitative similarity between fetal and adult hepatocytes’ functions and gene expression, mature adult hepatocytes’ responses to challenges widely differ quantitatively.^159^ Cholangiocytes are another important liver cells that mainly modify and transport hepatocyte-produced bile. Primary cholangiocytes are known to be sensitive to drugs, but involving them in liver drug screening models is restricted by their unavailability.^160^ Both iPSC-derived hepatocyte and cholangiocyte lineages showed immature phenotypes *in vitro*.^161^^,^^162^^,^^163^ Hence, finding tools to mature liver multilineage cells is necessary. Using liver ECMs as a maturing tool, rat liver ECM scaffolds induced iPSC-hepatocytes to express many signs of maturation, including high albumin/AFP ratios and mature markers (upregulation of ALB, GSTA1, and NDUFA3 and downregulation of CYP3A7 and AFP) compared to iPSC-hepatocytes cultured on Matrigel and PLLA-collagen type I scaffolds. The study showed that iPSC-hepatocytes presented better responsiveness to rifampicin (challenging the drug metabolism and the warfarin metabolism) and 3-methylcholanthrene (challenging the acetaminophen metabolism) up until day 14, qualitatively and quantitatively close to the responses of primary hepatocytes.^117^ This study and similar studies^164^^,^^165^ suggest that liver ECM derivatives (scaffolds/hydrogels) are excellent encapsulation hosts for iPSC-hepatocytes, magnifying their survival and functional maturity.

Excitingly, liver recellularization with stem cells or stem cell-derived committed cells is another approach to generate patient-specific liver models with mature multilineage cells and functional and transplantable grafts. In a recent example, recellularized ECMs of the human liver could promote the integration and maturation of hiPSC-cholangiocytes (repopulating the biliary tree ECMs), hiPSC-hepatocytes, hiPSC-vascular endothelial cells, hMSCs, human fibroblasts (repopulating the parenchymal space ECMs), and hiPSC-vascular endothelial cells (repopulating liver vasculature), showing excellent cell localization and attachment to liver ECM niches and mature functions of bile and albumin secretion, and A1AT and urea production similar to the function of human adult livers by comparison.^166^

### Liver organoids maturation

Current advances in developing stem cell-derived liver organoids hold a promising future for understanding liver development and diseases *in vitro*. However, mimicking liver spatiotemporal microenvironments and the cross-lineage relationship between liver parenchymal and non-parenchymal multilineage cells to grow mature liver organoids is still challenging.^167^^,^^168^ Although Matrigel takes the lead in encapsulating and promoting the self-organization of liver organoids,^169^ considering hydrogels with liver-specific biochemical cues might be necessary. Alternatively, liver ECM biomaterials are widely used to model and mature liver organoids.^170^^,^^171^^,^^172^^,^^173^^,^^174^^,^^175^^,^^176^ As a concept, a recent brilliant study reported that the accumulation of cell-deposited ECM secretum during the iPSCs to hepatocyte induction stages contributes to hepatocyte maturation. Briefly, the secreted ECM molecules of hepatic endoderm (205 proteins) and immature hepatocytes (226 proteins) during iPSCs induction stages to the mature hepatocytes stage are suggested to have played significant roles in accelerating hiPSCs differentiation and development into mature hepatic organoids in a microfluidic environment,^170^ another example of the importance of the existence of some ECM content (not only the induction medium) to advance organoid development. This study opens the door to the importance of investigating the potential of daily collecting and using differentiation stage-based conditioned media supplements to understand and accelerate organoid maturation. In one more example, porcine liver ECM hydrogels and a microfluidic system were used to mature hepatic organoids cocultured with HUVECs, wherein hepatic organoids had better urea production and albumin secretion and CYP3A4 activity, less apoptosis, enhanced cell-cell adhesion, and higher sensitivity to APAP-induced hepatotoxicity, compared to hepatic organoids encapsulated in collagen type I hydrogels, static conditions, and without vascularization.^175^ To our knowledge, no inclusive stem cell induction protocol can generate all liver multilineage cells. However, we hope to see the utilization of liver ECM biomaterials to generate liver multilineage cells and model mature liver organoids to help understand liver pathologies and disorders.

### Intestinal multilineage maturation

The lineup of the intestinal epithelial layer with millions of crypts and villi gives the gastrointestinal (GI) tract its unique architecture and function. The intestinal crypts serve as a niche for intestinal stem cells and a kick-off point for enterocytes, goblet cells, and Paneth cells, continuously born and migrating toward the top of the intestinal villi. The basal side of these epithelial cells is linked to the villi’s central cores, which possess arteries, veins, muscle strands, and lymphatic capillaries, linking the intestine to the circulation system. Interestingly, current advances showed the possibility of mimicking the 3D morphology of these crypt-villus structures using different modeling techniques. However, extra effort is needed to cellularize the lining and the cores of these intestinal 3D villi with more intestinal-inclusive multilineage cells to convey better intestinal function and hemostasis. The main hurdle toward achieving this goal comes from the extensive difficulty in maturing iPSC-derived intestinal multilineage cells, being the most feasible cell source, which were also reported to have immature phenotypes *in vitro*.^177^^,^^178^

Interestingly, ECM scaffolds and biomaterials from various GI tract regions are also used to model mature intestinal multilineage cells.^178^ For example, iPSC-derived intestinal progenitors and HUVECs were used to repopulate a segment of decellularized rat jejunum using a perfusion recellularization approach. Within 11 days, the hiPSC-derived progenitors had attached to different intestinal niches and differentiated into mature enteroendocrine cells and enterocytes. The repopulated model showed efficient glucose and fatty acid transfer *in vitro*, even without goblet cells and Paneth cell populations. When the recellularized model was implanted *in vivo*, more efficient glucose and fatty acids transfer was seen with the presence of all intestinal multilineage cells, including enterocytes, goblet cells, and Paneth cells (lining up the lumen) and intestinal subepithelial myofibroblast and smooth muscle cells (within the mesenchyme) and vascular cells (repopulating the vasculature and forming small blood vessels).^179^ More research can be done on recellularizing intestinal tissues for disease modeling or transplantation.

In another example, a vertical moving coaxial bioprinting approach was used to print functional microscale villi. To mimic the villus architecture, enterocytes were deposited through the nozzle’s shell (making the epithelial villus surface), and HUVECs were deposited through the nozzle’s core (making the vascularized villus core), both encapsulated in collagen-incorporated small intestine ECM hydrogels. Interestingly, enterocytes were mature, as evidenced by the substantial increase in the secretion of mucins and brush-border and digestive enzymes, as well as the increase in epithelial permeability, glucose uptake, and absorption functions, compared to enterocytes encapsulated in controls of collagen bioprinted scaffolds.^180^ However, due to precision limitations, there is still a long way to go to build complex crypt-villus using conventional methods like molding and bioprinting.

### Intestinal organoids maturation

Researchers can now mimic the GI tract multi-region microenvironments and grow hiPSCs-derived gastrointestinal organoids, such as esophageal, gastric, intestinal, and colonic organoids,^181^^,^^182^^,^^183^^,^^184^ recapitulating the morphology and function of the human GI tract. These developed models have unique region-specific morphologies, composing various types of gastrointestinal stem cells and their niches, with functioning multilineage cells, responding enteric nervous system, and active smooth muscle layers.^185^^,^^186^ However, the morphological maturity of gastrointestinal organoids that have recently advanced does not quite parallel their functional maturity outside *in vivo* systems. Attempts to better mature these models *in vitro* included using biomaterials incorporated with Matrigel or a cocktail of growth factors.^185^ However, their lack of tissue-specific instructive signals has shifted the preference toward ECM hydrogels.^178^ In a recent example, porcine stomach and intestine ECM hydrogels were used as alternatives to Matrigel to culture iPSC-derived gastric and intestinal organoids to overcome the batch-to-batch variation in Matrigel-encapsulated organoids but mainly to avoid possible Matrigel-related pathogenic transmission. Herein, both gastric organoids and intestinal organoids encapsulated in ECM hydrogels contained all their respective multilineage cells (including gastric mucous neck cells, chief cells, parietal cells, gastric stem cells, intestinal enterocytes, Paneth cells, goblet cells, enteroendocrine cells, and intestinal stem cells) and maintained the stem cells potent, while showing organotypic development and function and minimal morphological variations, but consistent, compared to Matrigel-encapsulated organoids.^75^

In the same fashion, different region-specific GI tract-derived ECMs have been used to grow mature or enhance the off-host functional maturity of patient-derived organoids such as gastric and small intestine organoids^187^ and duodenal, jejunal, and ileal organoids,^31^ wherein GI tract ECMs showed equivalent potential to encapsulate these organoids *in vitro* and encapsulate and deliver them *in vivo*, as alternatives to Matrigel. Taken together, GI tract organoid encapsulation in ECM hydrogels could boost their morphological and functional maturation in different hosting systems. Moreover, they were used to build encouraging and practical disease models, such as intestinal failure and short bowel syndrome models in the earlier examples. However, there needs to be more research using ECM hydrogels to encapsulate iPSC-derived GI tract organoids, which may represent a promising avenue for researchers to explore with a more rigorous assessment of functional maturity *in vitro*.

### Kidney multilineage maturation

Human kidneys comprise millions of long, permeable, multi-regional, multicellular tubular units called nephrons. The blood content reaching the nephron’s glomerular (a coiled network of highly permeable blood capillaries) is filtered at the renal capsule and allowed to pass through the nephron multi-regions lined up with epithelial multilineage cells, where the filtrate is further managed until executed out through the collecting duct. A mature podocyte lineup makes the glomerular and the nephron tubule interface. Podocytes selectively allow some blood content to pass through the nephron for extended filtration and detoxification.^188^ Kidney glomerular podocytes are indicated mature when the glomerular blood filtration barrier (nephrin) located close to the podocyte nucleus is pushed toward the plasma membrane upon maturity in conjugation with changes in podocytes’ polarity to maintain suitable glomerular slit diaphragm. At the same time, podocytes’ capacity for urinary clearance (albumin and inulin) and exogenous albumin uptake is exponentially increased.^189^ However, conveying neonatal, immortalized, or iPSC-derived podocytes to a high level of maturation is challenging, as they were reported to have immature phenotypes and a limited capacity to form interdigit networks *in vitro*.^190^^,^^191^^,^^192^^,^^193^ Among several approaches used to mature podocytes, such as laminin-coated surfaces^194^ and chemical conditioning,^195^^,^^196^ ECM biomaterials were also reported to enhance podocyte maturation. In one example, immortalized human podocytes grown on fibroblast-deposited ECMs showed high proliferation and significantly increased metabolic activity (p < 0.0001) up to 28 days. Podocytes also showed better morphology, mature cell-cell interconnection and interdigitations, and higher expression of synaptopodin and nephrin (Figure 4).^133^

Furthermore, several other nephrons’ lining epithelial cells’ morphological and functional maturation was improved using kidney ECM biomaterials. For example, human kidney peritubular microvascular endothelial cells (HKMECs, renal microcirculatory capillaries) cultured on kidney cortex ECM hydrogel were mature, evidenced by the significant increase in endothelial nitric oxide synthase while lowering proliferation and proteolytic activity, indicating better vascular integrity, less tight junction formation, and improved vessel permeability. However, HKMECs cultured on collagen type I and kidney ECM-collagen type I matrices displayed disturbed and immature phenotypes.^197^ In another interesting study, immature conditionally immortalized renal proximal tubular epithelial cells (ciPTECs) were used to recellularize 150 μm thick slices of a decellularized rat kidney, where ciPTEC gained better functional maturation within 7 days and showed high drug sensitivity to cisplatin and tenofovir toxicants within 24 h, demonstrating high suitability in building a mature nephrotoxicity drug testing model.^198^ Therefore, harnessing kidney ECM biomaterials to mature kidney nephron multilineage cells represents a promising venue for kidney 3D modeling. Region-specific kidney ECMs can be used as coating substrates for the microfluidic channels in the looked-for “nephron-on-a-chip” model^199^ or to substitute collagen in the “glomerulus-on-a-chip” model^200^ or to better model renal microcirculatory failure and other chronic kidney diseases.

### Kidney organoids maturation

Under appropriate spatiotemporal conditions, hPSC-derived kidney stem cells co-cultured with other assistive cell lineages evolve into kidney organoids containing several adequately vascularized and perfusable assemblies of the glomerulus, nephron, and collecting duct that mirror complex renal functions and respond to nephrotoxic agents.^105^^,^^201^^,^^202^ Still, more efforts are needed to bring the structure and function of kidney organoids to a comparable maturity to their *in vivo* counterparts.^203^^,^^204^ Many approaches were used in the attempts to mature kidney organoids, including vascularization,^201^^,^^205^^,^^206^ interconnected microwell arrays,^207^^,^^208^ bioreactors,^209^ and kidney ECM biomaterials.^76^^,^^210^ For example, porcine kidney ECM, used to grow hiPSC-derived kidney organoids, resulted in improving the glomerular structure and patterning, tubular structure and size, vascular areas and diameters, podocytes polarity, and proximal tubules polarization and enrichment with brush border markers and primary renal cilia. Kidney organoids also showed better proximal tubular reabsorption and dextran uptake after 48 h. High populations of podocytes, proximal tubule epithelial cells, the loop of Henle cells, and tubule progenitors were found in kidney organoids, which showed high similarity to their human adult equivalents. Interestingly, kidney organoids cultured in kidney ECMs had a lower number of off-target cell lineages compared to the high number of off-target cell lineages in Matrigel-cultured kidney organoids *in vitro*. Kidney organoids implanted in the immunodeficient mouse kidney capsule had better glomerulus basement membrane organization and mature podocytes-to-endothelial cells filtration barrier interface (Figure 4). Podocytes had secondary and tertiary foot processes interdigitated with glomerulus basement membrane showing similar structures to those of adult mouse kidneys.^76^

Using kidney-derived ECM biomaterials to encapsulate kidney organoids side by side with other methods can further mature kidney organoids. Nevertheless, building organoids with perfect nephron assemblies that permit blood content processed at the glomerulus to travel through the tubule requires better iPSC-to-kidney stem cell induction protocols that do not allow the generation of off-target cell lineages (reported to produce growth factors that disturb nephrogenesis^76^). Looking forward to methods that can guide nephron development extrinsically (such as light-assisted morphogenesis or driving kidney progenitors to travel in preselected nephron-like patterns) since recapitulating such a long, multiregional, and multicellular unit might be forever challenging.

## Advantages and disadvantages of ECM biomaterials

The objective to replicate precise native tissue properties encounters complexities due to the sophisticated nature of biological systems. Sourcing variability, batch-to-batch inconsistencies, and potential immunogenic responses associated with ECM biomaterials emphasize the need for careful examination. Optimizing the mechanical stability and prolonged efficacy of ECM biomaterials for specific applications demands further attention. During decellularization, ECM proteins are exposed to the risk of denaturation and misfolding. Despite efforts to avoid using irreversible denaturation inducers for decellularization and encourage the use of renaturation buffers and refolding techniques post-decellularization, tiny amounts of denatured and misfolded proteins may persist at some level, contributing to forming protein aggregates that may not be in favor of cellular activities, disturb cell communication, and may lead to tissue dysfunction in the long run. Regardless, many ECM-based products are well-established Food and Drug Administration-approved treatments and have been widely reported to have excellent therapeutic potential for tissue repair and regeneration.^211^^,^^212^^,^^213^

Overall, ECM biomaterials have been shown to provide a bioactive microenvironment that closely mimics native tissues, thereby promoting the development of functionally mature tissue constructs. Moreover, the immunomodulatory properties of ECM biomaterials further support their integration into different applications. Consolidating biocompatibility, bioactivity, and structural customization derived from various tissue sources presents the potential of ECM biomaterials to promote the growth of a wide range of multilineage cells, guide efficient differentiation, and promote tissue maturation. As evident from the reported findings, integrating ECM biomaterials into 3D modeling strategies can drive the field toward unique achievements, bringing about substantial improvements in regenerative medicine.

## Conclusion and future perspectives

Biomaterials have set the ground for the recent advances in 3D modeling, making the construction of sophisticated building blocks within reach. Further engaging biomaterials in applications like stem cell encapsulation, stemness preservation, fate determination, and tissue maturation necessitates biomaterials with a sufficient molecular inheritance that is wisely nominated and designed. However, developing dynamic ECM-mimicking biomaterials with comprehensive compositional values capable of kicking off different developmental stages during *in vitro* morphogenesis remains elusive. While the field has just realized the number of cell types and the importance of their collective roles in functionalizing adult human organs, most current induction protocols fail to generate all tissue-specific cell types. Thus, they are inadequate for bringing the unique multicellularity of human organs into current 3D models. What is further challenging is finding methods to mature stem cell-derived specialized cells to levels seen in their *in vivo* counterparts. As cells seem to go into clusters, assembling them into tissue-resembling architectures is extra challenging. Extrinsic interference and precise guidance of multilineage cell morphogenesis are required. However, interfering much is difficult, given our limited knowledge of the physical and biochemical cues needed to initiate certain morphogenic events within known time frames. One way to tackle these challenges is to use ECMs from nature to deliver extrinsic instructive signals known to support cell growth and expansion and contain regulatory molecules and growth factors that can preserve stem cell differentiation capacity and mediate the commitment and maturation of stem cell-derived cells and 3D models.

This review discussed the recent applications of decellularized ECM-derived biomaterials in driving stem cells into unique tissue-specific commitments toward functional organs and 3D models. Despite the ambiguity around their chemistry and compositional values, ECM biomaterials are currently leading the way in making physiologically relevant 3D models. However, more research decoding ECM compositions is needed to identify stem cells’ fate specification mediators and stemness caretakers, understand how off-target cells are reduced, and further engage lineage tracing techniques and fate probability measures to understand ECM biomaterials’ mechanism of action. For example, it will be revolutionary to understand MBV association with fate specification and cell maturation, just like their liquid-phase extracellular vesicle counterparts. Identifying cell-deposited ECM molecules engaged in different stem cell induction stages can also be promising to fast-track multilineage differentiation, increase throughput, and decrease batch-to-batch variations. Such research will one day enable bringing the values of the ECM to more modifiable biomaterials or forward-moving the usefulness of protein engineering end-products in regenerative medicine. We recommend using region-specific ECM biomaterials as the gold standard when developing new biomaterials for a region-specific application.

A snap into the near future, looking at cells morphing into organ-like structures as “migratory objects” will require the development of light-, magnet-, or robot-assisted morphogenesis platforms to spatially facilitate cell collective migration through the 3D matrix and enhance their adaptation of deterministic patterns. Looking at cells as “localizing objects” will require the development of light- or laser-assisted matrix manipulation platforms to spatially deform, crosslink, activate, and deactivate pre-implemented molecules or block/silence specific pathways/genes to assist cells in acquiring pattern-specific localization and collective morphologies. Coupling these techniques with live cell sequencing and fate identification, cell barcoding and tracking, and machine learning^214^^,^^215^^,^^216^^,^^217^^,^^218^^,^^219^ with tissue-specific algorithms will bring us to the era of fully automated morphogenesis, wherein building physiologically relevant organ-specific models will be, as a result, accessible.

## References

[1] Hofer M., Lutolf M.P. (2021). Engineering organoids. Nat. Rev. Mater..

[2] Nikolaev M., Mitrofanova O., Broguiere N., Geraldo S., Dutta D., Tabata Y., Elci B., Brandenberg N., Kolotuev I., Gjorevski N. (2020). Homeostatic mini-intestines through scaffold-guided organoid morphogenesis. Nature.

[3] Xiang Y., Tanaka Y., Cakir B., Patterson B., Kim K.Y., Sun P., Kang Y.J., Zhong M., Liu X., Patra P. (2019). hESC-Derived Thalamic Organoids Form Reciprocal Projections When Fused with Cortical Organoids. Cell Stem Cell.

[4] Brassard J.A., Lutolf M.P. (2019). Engineering Stem Cell Self-organization to Build Better Organoids. Cell Stem Cell.

[5] Lancaster M.A., Corsini N.S., Wolfinger S., Gustafson E.H., Phillips A.W., Burkard T.R., Otani T., Livesey F.J., Knoblich J.A. (2017). Guided self-organization and cortical plate formation in human brain organoids. Nat. Biotechnol..

[6] Ilina O., Friedl P. (2009). Mechanisms of collective cell migration at a glance. J. Cell Sci..

[7] Hakim V., Silberzan P. (2017). Collective cell migration: a physics perspective. Rep. Prog. Phys..

[8] Ladoux B., Mège R.M. (2017). Mechanobiology of collective cell behaviours. Nat. Rev. Mol. Cell Biol..

[9] Tarle V., Gauquelin E., Vedula S.R.K., D’Alessandro J., Lim C.T., Ladoux B., Gov N.S. (2017). Modeling collective cell migration in geometric confinement. Phys. Biol..

[10] Bich L., Pradeu T., Moreau J.F. (2019). Understanding Multicellularity: The Functional Organization of the Intercellular Space. Front. Physiol..

[11] Walma D.A.C., Yamada K.M. (2020). The extracellular matrix in development. Development.

[12] Rozario T., DeSimone D.W. (2010). The extracellular matrix in development and morphogenesis: A dynamic view. Dev. Biol..

[13] Bonnans C., Chou J., Werb Z. (2014). Remodelling the extracellular matrix in development and disease. Nat. Rev. Mol. Cell Biol..

[14] Ringer P., Colo G., Fässler R., Grashoff C. (2017). Sensing the mechano-chemical properties of the extracellular matrix. Matrix Biol..

[15] Urbanczyk M., Layland S.L., Schenke-Layland K. (2020). The role of extracellular matrix in biomechanics and its impact on bioengineering of cells and 3D tissues. Matrix Biol..

[16] Hussey G.S., Dziki J.L., Badylak S.F. (2018). Extracellular matrix-based materials for regenerative medicine. Nat. Rev. Mater..

[17] Meschiari C.A., Ero O.K., Pan H., Finkel T., Lindsey M.L. (2017). The impact of aging on cardiac extracellular matrix. Geroscience.

[18] Popova N.V., Jücker M. (2022). The Functional Role of Extracellular Matrix Proteins in Cancer. Cancers.

[19] Kratochvil M.J., Seymour A.J., Li T.L., Paşca S.P., Kuo C.J., Heilshorn S.C. (2019). Engineered materials for organoid systems. Nat. Rev. Mater..

[20] Watt F.M., Hogan B.L. (2000). Out of eden: Stem cells and their niches. Science.

[21] Morrison S.J., Spradling A.C. (2008). Stem Cells and Niches: Mechanisms That Promote Stem Cell Maintenance throughout Life. Cell.

[22] Frantz C., Stewart K.M., Weaver V.M. (2010). The extracellular matrix at a glance. J. Cell Sci..

[23] Theocharis A.D., Skandalis S.S., Gialeli C., Karamanos N.K. (2016). Extracellular matrix structure. Adv. Drug Deliv. Rev..

[24] Karamanos N.K., Theocharis A.D., Piperigkou Z., Manou D., Passi A., Skandalis S.S., Vynios D.H., Orian-Rousseau V., Ricard-Blum S., Schmelzer C.E.H. (2021). A guide to the composition and functions of the extracellular matrix. FEBS J..

[25] Hynes R.O., Naba A. (2012). Overview of the matrisome-An inventory of extracellular matrix constituents and functions. Cold Spring Harb. Perspect. Biol..

[26] Giobbe G.G., Crowley C., Luni C., Campinoti S., Khedr M., Kretzschmar K., De Santis M.M., Zambaiti E., Michielin F., Meran L. (2019). Extracellular matrix hydrogel derived from decellularized tissues enables endodermal organoid culture. Nat. Commun..

[27] de Sá Schiavo Matias G., da Silva Nunes Barreto R., Carreira A.C.O., Junior M.Y.N., Fratini P., Ferreira C.R., Miglino M.A. (2022). Proteomic profile of extracellular matrix from native and decellularized chorionic canine placenta. J. Proteomics.

[28] Bi H., Ye K., Jin S. (2020). Proteomic analysis of decellularized pancreatic matrix identifies collagen V as a critical regulator for islet organogenesis from human pluripotent stem cells. Biomaterials.

[29] Yuan M., Pai P.J., Liu X., Lam H., Chan B.P. (2018). Proteomic Analysis of Nucleus Pulposus Cell-derived Extracellular Matrix Niche and Its Effect on Phenotypic Alteration of Dermal Fibroblasts. Sci. Rep..

[30] Li Z., Tremmel D.M., Ma F., Yu Q., Ma M., Delafield D.G., Shi Y., Wang B., Mitchell S.A., Feeney A.K. (2021). Proteome-wide and matrisome-specific alterations during human pancreas development and maturation. Nat. Commun..

[31] Meran L., Massie I., Campinoti S., Weston A.E., Gaifulina R., Tullie L., Faull P., Orford M., Kucharska A., Baulies A. (2020). Engineering transplantable jejunal mucosal grafts using patient-derived organoids from children with intestinal failure. Nat. Med..

[32] Li M., Zhang T., Jiang J., Mao Y., Zhang A., Zhao J. (2019). ECM coating modification generated by optimized decellularization process improves functional behavior of BMSCs. Mater. Sci. Eng. C.

[33] Berger C., Bjørlykke Y., Hahn L., Mühlemann M., Kress S., Walles H., Luxenhofer R., Ræder H., Metzger M., Zdzieblo D. (2020). Matrix decoded – A pancreatic extracellular matrix with organ specific cues guiding human iPSC differentiation. Biomaterials.

[34] Leng L., Ma J., Sun X., Guo B., Li F., Zhang W., Chang M., Diao J., Wang Y., Wang W. (2020). Comprehensive proteomic atlas of skin biomatrix scaffolds reveals a supportive microenvironment for epidermal development. J. Tissue Eng..

[35] Mayorca-Guiliani A.E., Madsen C.D., Cox T.R., Horton E.R., Venning F.A., Erler J.T. (2017). ISDoT: In situ decellularization of tissues for high-resolution imaging and proteomic analysis of native extracellular matrix. Nat. Med..

[36] Chang C.W., Dalgliesh A.J., López J.E., Griffiths L.G. (2016). Cardiac extracellular matrix proteomics: Challenges, techniques, and clinical implications. Proteomics. Clin. Appl..

[37] Naba A., Clauser K.R., Mani D.R., Carr S.A., Hynes R.O. (2017). Quantitative proteomic profiling of the extracellular matrix of pancreatic islets during the angiogenic switch and insulinoma progression. Sci. Rep..

[38] Peng Y., Qing X., Lin H., Huang D., Li J., Tian S., Liu S., Lv X., Ma K., Li R. (2021). Decellularized Disc Hydrogels for hBMSCs tissue-specific differentiation and tissue regeneration. Bioact. Mater..

[39] Wang C., Yang X., Zhang X., Liu B., Liu W., Shen Y., Gao Z., Yin Q., Wang C., Zhou J. (2021). TMT-based quantitative proteome profiles reveal the memory function of a whole heart decellularized matrix for neural stem cell trans-differentiation into the cardiac lineage. Biomater. Sci..

[40] Gilpin S.E., Guyette J.P., Gonzalez G., Ren X., Asara J.M., Mathisen D.J., Vacanti J.P., Ott H.C. (2014). Perfusion decellularization of human and porcine lungs: Bringing the matrix to clinical scale. J. Heart Lung Transplant..

[41] Zhang R., Jiang J., Yu Y., Wang F., Gao N., Zhou Y., Wan X., Wang Z., Wei P., Mei J. (2021). Analysis of structural components of decellularized scaffolds in renal fibrosis. Bioact. Mater..

[42] Cocucci E., Meldolesi J. (2015). Ectosomes and exosomes: shedding the confusion between extracellular vesicles. Trends Cell Biol..

[43] Couch Y., Buzàs E.I., Di Vizio D., Gho Y.S., Harrison P., Hill A.F., Lötvall J., Raposo G., Stahl P.D., Théry C. (2021). A brief history of nearly EV-erything – The rise and rise of extracellular vesicles. J. Extracell. Vesicles.

[44] Hussey G.S., Pineda Molina C., Cramer M.C., Tyurina Y.Y., Tyurin V.A., Lee Y.C., El-Mossier S.O., Murdock M.H., Timashev P.S., Kagan V.E., Badylak S.F. (2020). Lipidomics and RNA sequencing reveal a novel subpopulation of nanovesicle within extracellular matrix biomaterials. Sci. Adv..

[45] Huleihel L., Hussey G.S., Naranjo J.D., Zhang L., Dziki J.L., Turner N.J., Stolz D.B., Badylak S.F. (2016). Matrix-bound nanovesicles within ECM bioscaffolds. Sci. Adv..

[46] Lenzini S., Debnath K., Joshi J.C., Wong S.W., Srivastava K., Geng X., Cho I.S., Song A., Bargi R., Lee J.C. (2021). Cell–Matrix Interactions Regulate Functional Extracellular Vesicle Secretion from Mesenchymal Stromal Cells. ACS Nano.

[47] Rilla K., Mustonen A.M., Arasu U.T., Härkönen K., Matilainen J., Nieminen P. (2019). Extracellular vesicles are integral and functional components of the extracellular matrix. Matrix Biol..

[48] Huleihel L., Bartolacci J.G., Dziki J.L., Vorobyov T., Arnold B., Scarritt M.E., Pineda Molina C., LoPresti S.T., Brown B.N., Naranjo J.D., Badylak S.F. (2017). Matrix-Bound Nanovesicles Recapitulate Extracellular Matrix Effects on Macrophage Phenotype. Tissue Eng. Part A.

[49] Cramer M., Pineda Molina C., Hussey G., Turnquist H.R., Badylak S.F. (2022). Transcriptomic Regulation of Macrophages by Matrix-Bound Nanovesicle-Associated Interleukin-33. Tissue Eng. Part A.

[50] Wang Z.-X., Luo Z.-W., Li F.-X.-Z., Cao J., Rao S.-S., Liu Y.-W., Wang Y.-Y., Zhu G.-Q., Gong J.-S., Zou J.-T. (2022). Aged bone matrix-derived extracellular vesicles as a messenger for calcification paradox. Nat. Commun..

[51] Bai C., Ren Q., Liu H., Li X., Guan W., Gao Y. (2021). miR-212/132-Enriched Extracellular Vesicles Promote Differentiation of Induced Pluripotent Stem Cells Into Pancreatic Beta Cells. Front. Cell Dev. Biol..

[52] Xu J., Du Y., Deng H. (2015). Direct lineage reprogramming: Strategies, mechanisms, and applications. Cell Stem Cell.

[53] Kim H., Song B.-W., Park S.-J., Choi S.W., Moon H., Hwang K.-C., Kang S.-W., Moon S.-H., Yang Y., Kwon I.C., Kim S.H. (2022). Ultraefficient extracellular vesicle guided direct reprogramming of fibroblasts into functional cardiomyocytes. Sci. Adv..

[54] Lin Z., McClure M.J., Zhao J., Ramey A.N., Asmussen N., Hyzy S.L., Schwartz Z., Boyan B.D. (2018). MicroRNA Contents in Matrix Vesicles Produced by Growth Plate Chondrocytes are Cell Maturation Dependent. Sci. Rep..

[55] Murry C.E., Keller G. (2008). Differentiation of Embryonic Stem Cells to Clinically Relevant Populations: Lessons from Embryonic Development. Cell.

[56] Watt F.M., Hogan B.L.M. (1999). Células troncos e seus nichos. Stem Cell Res. Ethics.

[57] Loh K.M., Lim B. (2011). A precarious balance: Pluripotency factors as lineage specifiers. Cell Stem Cell.

[58] T’Jonck W., Guilliams M., Bonnardel J. (2018). Niche signals and transcription factors involved in tissue-resident macrophage development. Cell. Immunol..

[59] Yamamizu K., Piao Y., Sharov A.A., Zsiros V., Yu H., Nakazawa K., Schlessinger D., Ko M.S.H. (2013). Identification of transcription factors for lineage-specific ESC differentiation. Stem Cell Rep..

[60] Semrau S., Goldmann J.E., Soumillon M., Mikkelsen T.S., Jaenisch R., Van Oudenaarden A. (2017). Dynamics of lineage commitment revealed by single-cell transcriptomics of differentiating embryonic stem cells. Nat. Commun..

[61] Ferraro F., Celso C.L., Scadden D., Meshorer E., Plath K., Meshorer E., Plath K. (2010). The Cell Biology of Stem Cells.

[62] Adams G.B., Martin R.P., Alley I.R., Chabner K.T., Cohen K.S., Calvi L.M., Kronenberg H.M., Scadden D.T. (2007). Therapeutic targeting of a stem cell niche. Nat. Biotechnol..

[63] Goel A.J., Rieder M.K., Arnold H.H., Radice G.L., Krauss R.S. (2017). Niche Cadherins Control the Quiescence-to-Activation Transition in Muscle Stem Cells. Cell Rep..

[64] Fuchs E., Tumbar T., Guasch G. (2004). Socializing with the neighbors: Stem cells and their niche. Cell.

[65] Fuchs E., Blau H.M. (2020). Tissue Stem Cells: Architects of Their Niches. Cell Stem Cell.

[66] Gattazzo F., Urciuolo A., Bonaldo P. (2014). Extracellular matrix: A dynamic microenvironment for stem cell niche. Biochim. Biophys. Acta.

[67] Eggenhofer E., Benseler V., Kroemer A., Popp F.C., Geissler E.K., Schlitt H.J., Baan C.C., Dahlke M.H., Hoogduijn M.J. (2012). Mesenchymal stem cells are short-lived and do not migrate beyond the lungs after intravenous infusion. Front. Immunol..

[68] Wang D., Zhu Y., Huang Y., Zhu J., Zhu B., Zhao Y., Lu Y., Wang Z., Guo Y. (2021). Pancreatic Extracellular Matrix/Alginate Hydrogels Provide a Supportive Microenvironment for Insulin-Producing Cells. ACS Biomater. Sci. Eng..

[69] Ota K.I. (2008). Fuel Cells: Past, Present and Future. IEEJ Trans. FM..

[70] Goh S.K., Olsen P., Banerjee I. (2013). Extracellular Matrix Aggregates from Differentiating Embryoid Bodies as a Scaffold to Support ESC Proliferation and Differentiation. PLoS One.

[71] Cesare E., Urciuolo A., Stuart H.T., Torchio E., Gesualdo A., Laterza C., Gagliano O., Martewicz S., Cui M., Manfredi A. (2022). 3D ECM-rich environment sustains the identity of naive human iPSCs. Cell Stem Cell.

[72] Yan Y., Martin L.M., Bosco D.B., Bundy J.L., Nowakowski R.S., Sang Q.-X.A., Li Y. (2015). Differential effects of acellular embryonic matrices on pluripotent stem cell expansion and neural differentiation. Biomaterials.

[73] Cesare E., Urciuolo A., Stuart H.T., Torchio E., Gesualdo A., Laterza C., Gagliano O., Martewicz S., Cui M., Manfredi A. (2022). 3D ECM-rich environment sustains the identity of naive human iPSCs. Cell Stem Cell.

[74] Li D., Chiu G., Lipe B., Hopkins R.A., Lillis J., Ashton J.M., Paul S., Aljitawi O.S. (2019). Decellularized Wharton jelly matrix: A biomimetic scaffold for ex vivo hematopoietic stem cell culture. Blood Adv..

[75] Kim S., Min S., Choi Y.S., Jo S.H., Jung J.H., Han K., Kim J., An S., Ji Y.W., Kim Y.G., Cho S.W. (2022). Tissue extracellular matrix hydrogels as alternatives to Matrigel for culturing gastrointestinal organoids. Nat. Commun..

[76] Kim J.W., Nam S.A., Yi J., Kim J.Y., Lee J.Y., Park S.Y., Sen T., Choi Y.M., Lee J.Y., Kim H.L. (2022). Kidney Decellularized Extracellular Matrix Enhanced the Vascularization and Maturation of Human Kidney Organoids. Adv. Sci..

[77] Tajima K., Yagi H., Morisaku T., Nishi K., Kushige H., Kojima H., Higashi H., Kuroda K., Kitago M., Adachi S. (2022). An organ-derived extracellular matrix triggers in situ kidney regeneration in a preclinical model. NPJ Regen. Med..

[78] Datta N., Pham Q.P., Sharma U., Sikavitsas V.I., Jansen J.A., Mikos A.G. (2006). In vitro generated extracellular matrix and fluid shear stress synergistically enhance 3D osteoblastic differentiation. Proc. Natl. Acad. Sci. USA.

[79] Pham Q.P., Kasper F.K., Scott Baggett L., Raphael R.M., Jansen J.A., Mikos A.G. (2008). The influence of an in vitro generated bone-like extracellular matrix on osteoblastic gene expression of marrow stromal cells. Biomaterials.

[80] Cheng H.W., Tsui Y.K., Cheung K.M.C., Chan D., Chan B.P. (2009). Decellularization of chondrocyte-encapsulated collagen microspheres: A three-dimensional model to study the effects of acellular matrix on stem cell fate. Tissue Eng. Part C Methods.

[81] Choi D.H., Suhaeri M., Hwang M.P., Kim I.H., Han D.K., Park K. (2014). Multi-lineage differentiation of human mesenchymal stromal cells on the biophysical microenvironment of cell-derived matrix. Cell Tissue Res..

[82] Chen G., Dong C., Yang L., Lv Y. (2015). 3D Scaffolds with Different Stiffness but the Same Microstructure for Bone Tissue Engineering. ACS Appl. Mater. Interfaces.

[83] Taylor B., Indano S., Yankannah Y., Patel P., Perez X.I., Freeman J. (2019). Decellularized Cortical Bone Scaffold Promotes Organized Neovascularization In Vivo. Tissue Eng. Part A.

[84] Santhakumar R., Vidyasekar P., Verma R.S. (2014). Cardiogel: A nano-matrix scaffold with potential application in cardiac regeneration using mesenchymal stem cells. PLoS One.

[85] Narayanan K., Lim V.Y., Shen J., Tan Z.W., Rajendran D., Luo S.C., Gao S., Wan A.C.A., Ying J.Y. (2014). Extracellular matrix-mediated differentiation of human embryonic stem cells: Differentiation to insulin-secreting beta cells. Tissue Eng. Part A.

[86] Duan Y., Liu Z., O’Neill J., Wan L.Q., Freytes D.O., Vunjak-Novakovic G. (2011). Hybrid gel composed of native heart matrix and collagen induces cardiac differentiation of human embryonic stem cells without supplemental growth factors. J. Cardiovasc. Transl. Res..

[87] Yu Y., Cui H., Zhang D., Liang B., Chai Y., Wen G. (2019). Human nail bed-derived decellularized scaffold regulates mesenchymal stem cells for nail plate regeneration. J. Tissue Eng. Regen. Med..

[88] da Mata Martins T.M., da Silva Cunha P., Rodrigues M.A., de Carvalho J.L., de Souza J.E., de Carvalho Oliveira J.A., Gomes D.A., de Goes A.M. (2020). Epithelial basement membrane of human decellularized cornea as a suitable substrate for differentiation of embryonic stem cells into corneal epithelial-like cells. Mater. Sci. Eng. C.

[89] Segel M., Neumann B., Hill M.F.E., Weber I.P., Viscomi C., Zhao C., Young A., Agley C.C., Thompson A.J., Gonzalez G.A. (2019). Niche stiffness underlies the ageing of central nervous system progenitor cells. Nature.

[90] Trentesaux C., Striedinger K., Pomerantz J.H., Klein O.D. (2020). From gut to glutes: The critical role of niche signals in the maintenance and renewal of adult stem cells. Curr. Opin. Cell Biol..

[91] Li J., Rao Z., Zhao Y., Xu Y., Chen L., Shen Z., Bai Y., Lin Z., Huang Q. (2020). A Decellularized Matrix Hydrogel Derived from Human Dental Pulp Promotes Dental Pulp Stem Cell Proliferation, Migration, and Induced Multidirectional Differentiation In Vitro. J. Endod..

[92] Scavuzzo M.A., Yang D., Borowiak M. (2017). Organotypic pancreatoids with native mesenchyme develop Insulin producing endocrine cells. Sci. Rep..

[93] Tan W.H., Gilmore E.C., Baris H.N. (2013).

[94] Lee C., Hu J., Ralls S., Kitamura T., Loh Y.P., Yang Y., Mukouyama Y.s., Ahn S. (2012). The Molecular Profiles of Neural Stem Cell Niche in the Adult Subventricular Zone. PLoS One.

[95] Choi H.-R., Byun S.Y., Kwon S.H., Park K.C. (2015). Niche interactions in epidermal stem cells. World J. Stem Cells.

[96] Bombelli S., Meregalli C., Scalia C., Bovo G., Torsello B., De Marco S., Cadamuro M., Viganò P., Strada G., Cattoretti G. (2018). Nephrosphere-Derived Cells Are Induced to Multilineage Differentiation when Cultured on Human Decellularized Kidney Scaffolds. Am. J. Pathol..

[97] Guyette J.P., Gilpin S.E., Charest J.M., Tapias L.F., Ren X., Ott H.C. (2014). Perfusion decellularization of whole organs. Nat. Protoc..

[98] Lu T.Y., Lin B., Kim J., Sullivan M., Tobita K., Salama G., Yang L. (2013). Repopulation of decellularized mouse heart with human induced pluripotent stem cell-derived cardiovascular progenitor cells. Nat. Commun..

[99] Bonandrini B., Figliuzzi M., Papadimou E., Morigi M., Perico N., Casiraghi F., Dipl C., Sangalli F., Conti S., Benigni A. (2014). Recellularization of well-preserved acellular kidney scaffold using embryonic stem cells. Tissue Eng. Part A.

[100] Maqueda M., Mosquera J.L., García-Arumí J., Veiga A., Duarri A. (2021). Repopulation of decellularized retinas with hiPSC-derived retinal pigment epithelial and ocular progenitor cells shows cell engraftment, organization and differentiation. Biomaterials.

[101] Uday Chandrika K., Tripathi R., Kameshwari Y., Rangaraj N., Mahesh Kumar J., Singh S. (2021). Refunctionalization of Decellularized Organ Scaffold of Pancreas by Recellularization: Whole Organ Regeneration into Functional Pancreas. Tissue Eng. Regen. Med..

[102] Lawson J.H., Glickman M.H., Ilzecki M., Jakimowicz T., Jaroszynski A., Peden E.K., Pilgrim A.J., Prichard H.L., Guziewicz M., Przywara S. (2016). Bioengineered human acellular vessels for dialysis access in patients with end-stage renal disease: Two phase 2 single-arm trials. Lancet.

[103] Kirkton R.D., Santiago-Maysonet M., Lawson J.H., Tente W.E., Dahl S.L.M., Niklason L.E., Prichard H.L. (2019). Erratum: Bioengineered human acellular vessels recellularize and evolve into living blood vessels after human implantation. Sci. Transl. Med..

[104] Siehler J., Blöchinger A.K., Meier M., Lickert H. (2021). Engineering islets from stem cells for advanced therapies of diabetes. Nat. Rev. Drug Discov..

[105] van den Berg C.W., Ritsma L., Avramut M.C., Wiersma L.E., van den Berg B.M., Leuning D.G., Lievers E., Koning M., Vanslambrouck J.M., Koster A.J. (2018). Renal Subcapsular Transplantation of PSC-Derived Kidney Organoids Induces Neo-vasculogenesis and Significant Glomerular and Tubular Maturation In Vivo. Stem Cell Rep..

[106] Escribá R., Ferrer-Lorente R., Raya Á. (2021). Inborn errors of metabolism: Lessons from iPSC models. Rev. Endocr. Metab. Disord..

[107] Vethe H., Bjørlykke Y., Ghila L.M., Paulo J.A., Scholz H., Gygi S.P., Chera S., Ræder H. (2017). Probing the missing mature β-cell proteomic landscape in differentiating patient iPSC-derived cells. Sci. Rep..

[108] Goversen B., van der Heyden M.A.G., van Veen T.A.B., de Boer T.P. (2018). The immature electrophysiological phenotype of iPSC-CMs still hampers in vitro drug screening: Special focus on IK1. Pharmacol. Ther..

[109] Alvarez-Dominguez J.R., Melton D.A. (2022). Cell maturation: Hallmarks, triggers, and manipulation. Cell.

[110] Lancaster M.A., Knoblich J.A. (2014). Organogenesisin a dish: Modeling development and disease using organoid technologies. Science.

[111] Lou Y.-R., Leung A.W. (2018). Next generation organoids for biomedical research and applications. Biotechnol. Adv..

[112] Takasato M., Er P.X., Chiu H.S., Maier B., Baillie G.J., Ferguson C., Parton R.G., Wolvetang E.J., Roost M.S., Chuva de Sousa Lopes S.M., Little M.H. (2015). Kidney organoids from human iPS cells contain multiple lineages and model human nephrogenesis. Nature.

[113] Vanslambrouck J.M., Wilson S.B., Tan K.S., Groenewegen E., Rudraraju R., Neil J., Lawlor K.T., Mah S., Scurr M., Howden S.E. (2022). Enhanced metanephric specification to functional proximal tubule enables toxicity screening and infectious disease modelling in kidney organoids. Nat. Commun..

[114] Aisenbrey E.A., Murphy W.L. (2020). Synthetic alternatives to Matrigel. Nat. Rev. Mater..

[115] Kozlowski M.T., Crook C.J., Ku H.T. (2021). Towards organoid culture without Matrigel. Commun. Biol..

[116] Block T., Creech J., da Rocha A.M., Marinkovic M., Ponce-Balbuena D., Jiménez-Vázquez E.N., Griffey S., Herron T.J. (2020). Human perinatal stem cell derived extracellular matrix enables rapid maturation of hiPSC-CM structural and functional phenotypes. Sci. Rep..

[117] Wang B., Jakus A.E., Baptista P.M., Soker S., Soto-Gutierrez A., Abecassis M.M., Shah R.N., Wertheim J.A. (2016). Functional Maturation of Induced Pluripotent Stem Cell Hepatocytes in Extracellular Matrix—A Comparative Analysis of Bioartificial Liver Microenvironments. Stem Cells Transl. Med..

[118] Schörnig M., Ju X., Fast L., Ebert S., Weigert A., Kanton S., Schaffer T., Nadif Kasri N., Treutlein B., Peter B.M. (2021). Comparison of induced neurons reveals slower structural and functional maturation in humans than in apes. Elife.

[119] Roth J.G., Huang M.S., Li T.L., Feig V.R., Jiang Y., Cui B., Greely H.T., Bao Z., Paşca S.P., Heilshorn S.C. (2021). Advancing models of neural development with biomaterials. Nat. Rev. Neurosci..

[120] Sood D., Cairns D.M., Dabbi J.M., Ramakrishnan C., Deisseroth K., Black L.D., Santaniello S., Kaplan D.L. (2019). Functional maturation of human neural stem cells in a 3D bioengineered brain model enriched with fetal brain-derived matrix. Sci. Rep..

[121] Xu Y., Zhou J., Liu C., Zhang S., Gao F., Guo W., Sun X., Zhang C., Li H., Rao Z. (2021). Understanding the role of tissue-specific decellularized spinal cord matrix hydrogel for neural stem/progenitor cell microenvironment reconstruction and spinal cord injury. Biomaterials.

[122] Moore S., Meschkat M., Ruhwedel T., Trevisiol A., Tzvetanova I.D., Battefeld A., Kusch K., Kole M.H.P., Strenzke N., Möbius W. (2020). A role of oligodendrocytes in information processing. Nat. Commun..

[123] van Tilborg E., Heijnen C.J., Benders M.J., van Bel F., Fleiss B., Gressens P., Nijboer C.H. (2016). Impaired oligodendrocyte maturation in preterm infants: Potential therapeutic targets. Prog. Neurobiol..

[124] Lai B.Q., Bai Y.R., Han W.T., Zhang B., Liu S., Sun J.H., Liu J.L., Li G., Zeng X., Ding Y. (2022). Construction of a niche-specific spinal white matter-like tissue to promote directional axon regeneration and myelination for rat spinal cord injury repair. Bioact. Mater..

[125] Chen S., Du Z., Zou J., Qiu S., Rao Z., Liu S., Sun X., Xu Y., Zhu Q., Liu X. (2019). Promoting Neurite Growth and Schwann Cell Migration by the Harnessing Decellularized Nerve Matrix onto Nanofibrous Guidance. ACS Appl. Mater. Interfaces.

[126] Li R., Xu J., Rao Z., Deng R., Xu Y., Qiu S., Long H., Zhu Q., Liu X., Bai Y., Quan D. (2021). Facilitate Angiogenesis and Neurogenesis by Growth Factors Integrated Decellularized Matrix Hydrogel. Tissue Eng. Part A.

[127] van der Merwe Y., Faust A.E., Sakalli E.T., Westrick C.C., Hussey G., Chan K.C., Conner I.P., Fu V.L.N., Badylak S.F., Steketee M.B. (2019). Matrix-bound nanovesicles prevent ischemia-induced retinal ganglion cell axon degeneration and death and preserve visual function. Sci. Rep..

[128] Di Lullo E., Kriegstein A.R. (2017). The use of brain organoids to investigate neural development and disease. Nat. Rev. Neurosci..

[129] Kim J., Sullivan G.J., Park I.H. (2021). How well do brain organoids capture your brain?. iScience.

[130] Gordon A., Yoon S.J., Tran S.S., Makinson C.D., Park J.Y., Andersen J., Valencia A.M., Horvath S., Xiao X., Huguenard J.R. (2021). Long-term maturation of human cortical organoids matches key early postnatal transitions. Nat. Neurosci..

[131] Cho A.N., Jin Y., An Y., Kim J., Choi Y.S., Lee J.S., Kim J., Choi W.Y., Koo D.J., Yu W. (2021). Microfluidic device with brain extracellular matrix promotes structural and functional maturation of human brain organoids. Nat. Commun..

[132] Li Z., Zhao Y., Lv X., Deng Y. (2023). Integrated brain on a chip and automated organ-on-chips systems. Interdiscip. Med..

[133] Satyam A., Tsokos M.G., Tresback J.S., Zeugolis D.I., Tsokos G.C. (2020). Cell-Derived Extracellular Matrix-Rich Biomimetic Substrate Supports Podocyte Proliferation, Differentiation, and Maintenance of Native Phenotype. Adv. Funct. Mater..

[134] Robertson C., Tran D.D., George S.C. (2013). Concise review: Maturation phases of human pluripotent stem cell-derived cardiomyocytes. Stem Cell..

[135] Laflamme M.A., Murry C.E. (2011). Heart regeneration. Nature.

[136] Ronaldson-Bouchard K., Ma S.P., Yeager K., Chen T., Song L., Sirabella D., Morikawa K., Teles D., Yazawa M., Vunjak-Novakovic G. (2018). Advanced maturation of human cardiac tissue grown from pluripotent stem cells. Nature.

[137] Kolanowski T.J., Busek M., Schubert M., Dmitrieva A., Binnewerg B., Pöche J., Fisher K., Schmieder F., Grünzner S., Hansen S. (2020). Enhanced structural maturation of human induced pluripotent stem cell-derived cardiomyocytes under a controlled microenvironment in a microfluidic system. Acta Biomater..

[138] Li H., Ye W., Yu B., Yan X., Lin Y., Zhan J., Chen P., Song X., Yang P., Cai Y. (2023). Supramolecular Assemblies of Glycopeptides Enhance Gap Junction Maturation of Human Induced Pluripotent Stem Cell-Derived Cardiomyocytes via Inducing Spheroids Formation to Optimize Cardiac Repair. Adv. Healthc. Mater..

[139] Karbassi E., Fenix A., Marchiano S., Muraoka N., Nakamura K., Yang X., Murry C.E. (2020). Cardiomyocyte maturation: advances in knowledge and implications for regenerative medicine. Nat. Rev. Cardiol..

[140] Nunes S.S., Miklas J.W., Liu J., Aschar-Sobbi R., Xiao Y., Zhang B., Jiang J., Massé S., Gagliardi M., Hsieh A. (2013). Biowire: A platform for maturation of human pluripotent stem cell-derived cardiomyocytes. Nat. Methods.

[141] Scuderi G.J., Butcher J. (2017). Naturally engineered maturation of cardiomyocytes. Front. Cell Dev. Biol..

[142] Almeida H.V., Tenreiro M.F., Louro A.F., Abecasis B., Santinha D., Calmeiro T., Fortunato E., Ferreira L., Alves P.M., Serra M. (2021). Human Extracellular-Matrix Functionalization of 3D hiPSC-Based Cardiac Tissues Improves Cardiomyocyte Maturation. ACS Appl. Bio Mater..

[143] Ozcebe S.G., Bahcecioglu G., Yue X.S., Zorlutuna P. (2021). Effect of cellular and ECM aging on human iPSC-derived cardiomyocyte performance, maturity and senescence. Biomaterials.

[144] Wortham M., Sander M. (2021). Transcriptional mechanisms of pancreatic β-cell maturation and functional adaptation. Trends Endocrinol. Metab..

[145] Velazco-Cruz L., Goedegebuure M.M., Millman J.R. (2020). Advances Toward Engineering Functionally Mature Human Pluripotent Stem Cell-Derived β Cells. Front. Bioeng. Biotechnol..

[146] Parent A.V., Ashe S., Nair G.G., Li M.L., Chavez J., Liu J.S., Zhong Y., Streeter P.R., Hebrok M. (2022). Development of a scalable method to isolate subsets of stem cell-derived pancreatic islet cells. Stem Cell Rep..

[147] Kim M., Jang J. (2021). Construction of 3D hierarchical tissue platforms for modeling diabetes. APL Bioeng..

[148] Hunckler M.D., García A.J. (2020). Engineered Biomaterials for Enhanced Function of Insulin-Secreting β-Cell Organoids. Adv. Funct. Mater..

[149] Patel S.N., Mathews C.E., Chandler R., Stabler C.L. (2022). The foundation for engineering a pancreatic islet niche. Front. Endocrinol..

[150] Hwang D.G., Jo Y., Kim M., Yong U., Cho S., Choi Y.M., Kim J., Jang J. (2021). A 3D bioprinted hybrid encapsulation system for delivery of human pluripotent stem cell-derived pancreatic islet-like aggregates. Biofabrication.

[151] Banerjee I. (2021). Strategies for Vascularizing Pancreatic Islets and Stem Cell–Derived Islet Organoids. Curr. Transplant. Rep..

[152] Kim J., Shim I.K., Hwang D.G., Lee Y.N., Kim M., Kim H., Kim S.W., Lee S., Kim S.C., Cho D.W., Jang J. (2019). 3D cell printing of islet-laden pancreatic tissue-derived extracellular matrix bioink constructs for enhancing pancreatic functions. J. Mater. Chem. B.

[153] Zhang X., Ma Z., Song E., Xu T. (2022). Islet organoid as a promising model for diabetes. Protein Cell.

[154] Bi H., Karanth S.S., Ye K., Stein R., Jin S. (2020). Decellularized Tissue Matrix Enhances Self-Assembly of Islet Organoids from Pluripotent Stem Cell Differentiation. ACS Biomater. Sci. Eng..

[155] Daly A.C., Riley L., Segura T., Burdick J.A. (2020). Hydrogel microparticles for biomedical applications. Nat. Rev. Mater..

[156] Vargas-Valderrama A., Messina A., Mitjavila-Garcia M.T., Guenou H. (2020). The endothelium, a key actor in organ development and hPSC-derived organoid vascularization. J. Biomed. Sci..

[157] Yin J., Meng H., Lin J., Ji W., Xu T., Liu H. (2022). Pancreatic islet organoids - on - a - chip : how far have we gone. J. Nanobiotechnol..

[158] Pierzynowski S.G., Gregory P.C., Filip R., Woliński J., Pierzynowska K.G. (2018). Glucose homeostasis dependency on acini–islet–acinar (AIA) axis communication: a new possible pathophysiological hypothesis regarding diabetes mellitus. Nutr. Diabetes.

[159] Rowe C., Gerrard D.T., Jenkins R., Berry A., Durkin K., Sundstrom L., Goldring C.E., Park B.K., Kitteringham N.R., Hanley K.P., Hanley N.A. (2013). Proteome-wide analyses of human hepatocytes during differentiation and dedifferentiation. Hepatology.

[160] Wang Z., Faria J., van der Laan L.J.W., Penning L.C., Masereeuw R., Spee B. (2022). Human Cholangiocytes Form a Polarized and Functional Bile Duct on Hollow Fiber Membranes. Front. Bioeng. Biotechnol..

[161] Yiangou L., Ross A.D.B., Goh K.J., Vallier L. (2018). Human Pluripotent Stem Cell-Derived Endoderm for Modeling Development and Clinical Applications. Cell Stem Cell.

[162] Kunst R.F., Niemeijer M., van der Laan L.J.W., Spee B., van de Graaf S.F.J. (2020). From fatty hepatocytes to impaired bile flow: Matching model systems for liver biology and disease. Biochem. Pharmacol..

[163] ter Braak B., Niemeijer M., Boon R., Parmentier C., Baze A., Richert L., Huppelschoten S., Wink S., Verfaillie C., van de Water B. (2021). Systematic transcriptome-based comparison of cellular adaptive stress response activation networks in hepatic stem cell-derived progeny and primary human hepatocytes. Toxicol. Vitr..

[164] Jaramillo M., Yeh H., Yarmush M.L., Uygun B.E. (2018). Decellularized human liver extracellular matrix (hDLM)-mediated hepatic differentiation of human induced pluripotent stem cells (hIPSCs). J. Tissue Eng. Regen. Med..

[165] Lee J.S., Shin J., Park H.M., Kim Y.G., Kim B.G., Oh J.W., Cho S.W. (2014). Liver extracellular matrix providing dual functions of two-dimensional substrate coating and three-dimensional injectable hydrogel platform for liver tissue engineering. Biomacromolecules.

[166] Takeishi K., Collin de l’Hortet A., Wang Y., Handa K., Guzman-Lepe J., Matsubara K., Morita K., Jang S., Haep N., Florentino R.M. (2020). Assembly and Function of a Bioengineered Human Liver for Transplantation Generated Solely from Induced Pluripotent Stem Cells. Cell Rep..

[167] Velazquez J.J., LeGraw R., Moghadam F., Tan Y., Kilbourne J., Maggiore J.C., Hislop J., Liu S., Cats D., Chuva de Sousa Lopes S.M. (2021). Gene Regulatory Network Analysis and Engineering Directs Development and Vascularization of Multilineage Human Liver Organoids. Cell Syst..

[168] Brooks A., Liang X., Zhang Y., Zhao C.X., Roberts M.S., Wang H., Zhang L., Crawford D.H.G. (2021). Liver organoid as a 3D in vitro model for drug validation and toxicity assessment. Pharmacol. Res..

[169] Sun L., Hui L. (2020). Progress in human liver organoids. J. Mol. Cell Biol..

[170] Michielin F., Giobbe G.G., Luni C., Hu Q., Maroni I., Orford M.R., Manfredi A., Di Filippo L., David A.L., Cacchiarelli D. (2020). The Microfluidic Environment Reveals a Hidden Role of Self-Organizing Extracellular Matrix in Hepatic Commitment and Organoid Formation of hiPSCs. Cell Rep..

[171] Saheli M., Sepantafar M., Pournasr B., Farzaneh Z., Vosough M., Piryaei A., Baharvand H. (2018). Three-dimensional liver-derived extracellular matrix hydrogel promotes liver organoids function. J. Cell. Biochem..

[172] Morales D.X., Grineski S.E. (2016). 乳鼠心肌提取 HHS Public Access. Physiol. Behav..

[173] Willemse J., van Tienderen G., van Hengel E., Schurink I., van der Ven D., Kan Y., de Ruiter P., Rosmark O., Westergren-Thorsson G., Schneeberger K. (2022). Hydrogels derived from decellularized liver tissue support the growth and differentiation of cholangiocyte organoids. Biomaterials.

[174] Caralt M., Velasco E., Lanas A., Baptista P.M. (2014). Liver bioengineering: From the stage of liver decellularized matrix to the multiple cellular actors and bioreactor special effects. Organogenesis.

[175] Jin Y., Kim J., Lee J.S., Min S., Kim S., Ahn D.H., Kim Y.G., Cho S.W. (2018). Vascularized Liver Organoids Generated Using Induced Hepatic Tissue and Dynamic Liver-Specific Microenvironment as a Drug Testing Platform. Adv. Funct. Mater..

[176] Roos F.J.M., Wu H., Willemse J., Lieshout R., Albarinos L.A.M., Kan Y.Y., Poley J.W., Bruno M.J., de Jonge J., Bártfai R. (2021). Cholangiocyte organoids from human bile retain a local phenotype and can repopulate bile ducts in vitro. Clin. Transl. Med..

[177] Wells J.M., Spence J.R. (2014). How to make an intestine. Dev.

[178] Tullie L., Jones B.C., De Coppi P., Li V.S.W. (2022). Building gut from scratch — progress and update of intestinal tissue engineering. Nat. Rev. Gastroenterol. Hepatol..

[179] Kitano K., Schwartz D.M., Zhou H., Gilpin S.E., Wojtkiewicz G.R., Ren X., Sommer C.A., Capilla A.V., Mathisen D.J., Goldstein A.M. (2017). Bioengineering of functional human induced pluripotent stem cell-derived intestinal grafts. Nat. Commun..

[180] Kim W., Kim G.H. (2020). An intestinal model with a finger-like villus structure fabricated using a bioprinting process and collagen/SIS-based cell-laden bioink. Theranostics.

[181] Kasagi Y., Chandramouleeswaran P.M., Whelan K.A., Tanaka K., Giroux V., Sharma M., Wang J., Benitez A.J., DeMarshall M., Tobias J.W. (2018). The Esophageal Organoid System Reveals Functional Interplay Between Notch and Cytokines in Reactive Epithelial Changes. Cmgh.

[182] McCracken K.W., Catá E.M., Crawford C.M., Sinagoga K.L., Schumacher M., Rockich B.E., Tsai Y.H., Mayhew C.N., Spence J.R., Zavros Y., Wells J.M. (2014). Modelling human development and disease in pluripotent stem-cell-derived gastric organoids. Nature.

[183] Dekkers J.F., Wiegerinck C.L., De Jonge H.R., Bronsveld I., Janssens H.M., De Winter-De Groot K.M., Brandsma A.M., De Jong N.W.M., Bijvelds M.J.C., Scholte B.J. (2013). A functional CFTR assay using primary cystic fibrosis intestinal organoids. Nat. Med..

[184] Crespo M., Vilar E., Tsai S.Y., Chang K., Amin S., Srinivasan T., Zhang T., Pipalia N.H., Chen H.J., Witherspoon M. (2017). Colonic organoids derived from human induced pluripotent stem cells for modeling colorectal cancer and drug testing. Nat. Med..

[185] Hirota A., AlMusawi S., Nateri A.S., Ordóñez-Morán P., Imajo M. (2021). Biomaterials for intestinal organoid technology and personalized disease modeling. Acta Biomater..

[186] Kim G.A., Spence J.R., Takayama S. (2017). Bioengineering for intestinal organoid cultures. Curr. Opin. Biotechnol..

[187] Giobbe G.G., Crowley C., Luni C., Campinoti S., Khedr M., Kretzschmar K., De Santis M.M., Zambaiti E., Michielin F., Meran L. (2019). Extracellular matrix hydrogel derived from decellularized tissues enables endodermal organoid culture. Nat. Commun..

[188] Legallais C., Kim D., Mihaila S.M., Mihajlovic M., Figliuzzi M., Bonandrini B., Salerno S., Yousef Yengej F.A., Rookmaaker M.B., Sanchez Romero N. (2018). Bioengineering Organs for Blood Detoxification. Adv. Healthc. Mater..

[189] Musah S., Mammoto A., Ferrante T.C., Jeanty S.S.F., Hirano-Kobayashi M., Mammoto T., Roberts K., Chung S., Novak R., Ingram M. (2017). Mature induced-pluripotent-stem-cell-derived human podocytes reconstitute kidney glomerular-capillary-wall function on a chip. Nat. Biomed. Eng..

[190] Song B., Smink A.M., Jones C.V., Callaghan J.M., Firth S.D., Bernard C.A., Laslett A.L., Kerr P.G., Ricardo S.D. (2012). The Directed Differentiation of Human iPS Cells into Kidney Podocytes. PLoS One.

[191] Shankar A.S., Hoorn E.J., Gribnau J., Baan C.C., Hoogduijn M.J. (2019). Current State of Renal Regenerative Therapies. Transplantation.

[192] Nam S.A., Seo E., Kim J.W., Kim H.W., Kim H.L., Kim K., Kim T.M., Ju J.H., Gomez I.G., Uchimura K. (2019). Graft immaturity and safety concerns in transplanted human kidney organoids. Exp. Mol. Med..

[193] Yoshimura Y., Nishinakamura R. (2019). Podocyte development, disease, and stem cell research. Kidney Int..

[194] Musah S., Dimitrakakis N., Camacho D.M., Church G.M., Ingber D.E. (2018). Directed differentiation of human induced pluripotent stem cells into mature kidney podocytes and establishment of a Glomerulus Chip. Nat. Protoc..

[195] Qian T., Hernday S.E., Bao X., Olson W.R., Panzer S.E., Shusta E.V., Palecek S.P. (2019). Directed Differentiation of Human Pluripotent Stem Cells to Podocytes under Defined Conditions. Sci. Rep..

[196] Burt M., Bhattachaya R., Okafor A.E., Musah S. (2020). Guided differentiation of mature kidney podocytes from human induced pluripotent stem cells under chemically defined conditions. J. Vis. Exp..

[197] Nagao R.J., Xu J., Luo P., Xue J., Wang Y., Kotha S., Zeng W., Fu X., Himmelfarb J., Zheng Y. (2016). Decellularized Human Kidney Cortex Hydrogels Enhance Kidney Microvascular Endothelial Cell Maturation and Quiescence. Tissue Eng. Part A.

[198] Fedecostante M., Westphal K.G.C., Buono M.F., Sanchez Romero N., Wilmer M.J., Kerkering J., Baptista P.M., Hoenderop J.G., Masereeuw R. (2018). Recellularized native kidney scaffolds as a novel tool in nephrotoxicity screening s. Drug Metab. Dispos..

[199] Ashammakhi N., Wesseling-Perry K., Hasan A., Elkhammas E., Zhang Y.S. (2018). Kidney-on-a-chip: untapped opportunities. Kidney Int..

[200] Petrosyan A., Cravedi P., Villani V., Angeletti A., Manrique J., Renieri A., De Filippo R.E., Perin L., Da Sacco S. (2019). A glomerulus-on-a-chip to recapitulate the human glomerular filtration barrier. Nat. Commun..

[201] Homan K.A., Gupta N., Kroll K.T., Kolesky D.B., Skylar-Scott M., Miyoshi T., Mau D., Valerius M.T., Ferrante T., Bonventre J.V. (2019). Flow-enhanced vascularization and maturation of kidney organoids in vitro. Nat. Methods.

[202] Garreta E., Nauryzgaliyeva Z., Montserrat N. (2021). Human induced pluripotent stem cell-derived kidney organoids toward clinical implementations. Curr. Opin. Biomed. Eng..

[203] Morizane R., Lam A.Q., Freedman B.S., Kishi S., Valerius M.T., Bonventre J.V. (2015). Nephron organoids derived from human pluripotent stem cells model kidney development and injury. Nat. Biotechnol..

[204] Nishinakamura R. (2019). Human kidney organoids: progress and remaining challenges. Nat. Rev. Nephrol..

[205] Lim D.S., Jackson J.D., Atala A., Yoo J.J. (2022). Leading Approaches to Vascularize Kidney Constructs in Tissue Engineering. Engineering.

[206] Koning M., van den Berg C.W., Rabelink T.J. (2020). Stem cell-derived kidney organoids: engineering the vasculature. Cell. Mol. Life Sci..

[207] Czerniecki S.M., Cruz N.M., Harder J.L., Menon R., Annis J., Otto E.A., Gulieva R.E., Islas L.V., Kim Y.K., Tran L.M. (2018). High-Throughput Screening Enhances Kidney Organoid Differentiation from Human Pluripotent Stem Cells and Enables Automated Multidimensional Phenotyping. Cell Stem Cell.

[208] Glass N.R., Takasako M., Er P.X., Titmarsh D.M., Hidalgo A., Wolvetang E.J., Little M.H., Cooper-White J.J. (2020). Multivariate patterning of human pluripotent cells under perfusion reveals critical roles of induced paracrine factors in kidney organoid development. Sci. Adv..

[209] Przepiorski A., Sander V., Tran T., Hollywood J.A., Sorrenson B., Shih J.H., Wolvetang E.J., McMahon A.P., Holm T.M., Davidson A.J. (2018). A Simple Bioreactor-Based Method to Generate Kidney Organoids from Pluripotent Stem Cells. Stem Cell Rep..

[210] Ding B., Sun G., Liu S., Peng E., Wan M., Chen L., Jackson J., Atala A. (2020). Three-dimensional renal organoids from whole kidney cells: Generation, optimization, and potential application in nephrotoxicology in vitro. Cell Transplant..

[211] Parmaksiz M., Dogan A., Odabas S., Elçin A.E., Elçin Y.M. (2016). Clinical applications of decellularized extracellular matrices for tissue engineering and regenerative medicine. Biomed. Mater..

[212] Zhang X., Chen X., Hong H., Hu R., Liu J., Liu C. (2022). Decellularized extracellular matrix scaffolds: Recent trends and emerging strategies in tissue engineering. Bioact. Mater..

[213] Wang B., Qinglai T., Yang Q., Li M., Zeng S., Yang X., Xiao Z., Tong X., Lei L., Li S. (2022). Functional acellular matrix for tissue repair. Mater. Today Bio.

[214] Kebschull J.M., Zador A.M. (2018). Cellular barcoding: lineage tracing, screening and beyond. Nat. Methods.

[215] Toda S., McKeithan W.L., Hakkinen T.J., Lopez P., Klein O.D., Lim W.A. (2020). Engineering synthetic morphogen systems that can program multicellular patterning. Science.

[216] Ebrahimkhani M.R., Levin M. (2021). Synthetic living machines: A new window on life. iScience.

[217] Kourti D., Kanioura A., Chatzichristidi M., Beltsios K.G., Kakabakos S.E., Petrou P.S. (2022). Photopatternable materials for guided cell adhesion and growth. Eur. Polym. J..

[218] Anand G., Megale H.C., Murphy S.H., Weis T., Lin Z., He Y., Wang X., Liu J. (2023). Controlling organoid symmetry breaking uncovers an excitable system underlying human axial elongation. Cell.

[219] Özelçi E., Mailand E., Rüegg M., Oates A.C., Sakar M.S. (2022). Deconstructing body axis morphogenesis in zebrafish embryos using robot-assisted tissue micromanipulation. bioRxiv.

